# Causal effects of stimulus-induced blood cell phenotypes on the risk of primary knee osteoarthritis: A two-sample Mendelian randomization study

**DOI:** 10.1097/MD.0000000000045999

**Published:** 2025-11-14

**Authors:** Dongxiao Li, Junhong Gan, Xiaomin Li, Lin Xiao, Hongli Teng, Shihai Xiao, Ben Liu, Kun Chen, Longpu Deng

**Affiliations:** a Department of Pain, Guangxi International Zhuang Medical Hospital Affiliated to Guangxi University of Chinese Medicine, Nanning, China; b Guangxi University of Chinese Medicine, Nanning, China; c Department of Traditional Chinese Medicine, Beihai People’s Hospital, Beihai, China.

**Keywords:** blood cell phenotype, immune stimulation, Mendelian randomization, Primary knee osteoarthritis

## Abstract

Primary knee osteoarthritis (PKOA) is a common degenerative joint disorder, where immune-mediated inflammation plays a key role in its onset and progression. However, the causal relationship between blood cell functional states under immune stimuli and PKOA risk has not been systematically evaluated. We conducted a 2-sample Mendelian randomization analysis to explore the causal effects of immune-related blood cell phenotypes on PKOA risk. Exposure data were derived from a GWAS by Homilius et al. (2024, Nature Genetics) involving 1312 European blood donors. Outcome data came from the PKOA GWAS in the FinnGen R12 cohort (23,582 cases and 438,992 controls). Causal estimates were calculated using inverse-variance weighted (IVW), Mendelian randomization-Egger regression, and the weighted median method. Four phenotypes were significantly associated with PKOA risk after correction for multiple testing: red blood cell membrane stability in response to rotenone, neutrophil volumetric and structural changes in response to Pam3CSK4, baseline morphological variations of an unclassified immune cell population, and structural remodeling of leukocytes in response to Pam3CSK4. Sensitivity analyses confirmed the robustness of these findings with no evidence of substantial pleiotropy or heterogeneity. This study is the first to explore causal links between immune-related blood cell phenotypes and PKOA risk. These findings suggest that immune functional phenotypes may serve as potential biomarkers for early detection and targeted intervention in PKOA, but further validation is required. Future research should include multi-ethnic validation and functional studies to confirm these biomarkers and assess their clinical applicability.

## 1. Introduction

PKOA is a common chronic degenerative disease that primarily affects the articular cartilage of the knee and surrounding tissues, with joint pain and restricted mobility as its hallmark clinical manifestations.^[1]^ In recent decades, the incidence of PKOA has been steadily increasing in parallel with global population aging, leading to a substantial rise in disability burden and making PKOA an important public health concern for middle-aged and older adults.^[2]^ Notably, the risk of PKOA among younger individuals is also on the rise,^[3]^ further complicating disease prevention and management. This trend underscores the urgency of advancing in-depth research into PKOA.

The pathogenesis of PKOA is multifactorial, involving mechanical loading, genetic susceptibility, metabolic dysregulation, and immune-mediated inflammation.^[4,5]^ In recent years, chronic low-grade inflammation and immune cell activation have increasingly been recognized as key drivers of PKOA onset and progression.^[6,7]^ Peripheral blood cells, which serve as a critical reflection of systemic immune status, play an active role in cytokine release, synovial microenvironment modulation, and tissue repair.^[8]^ However, direct evidence for their causal role in PKOA etiology remains limited.

Conventional studies of blood cell phenotypes have predominantly focused on static measurements, such as absolute counts or proportions, which fail to fully capture functional behavioral differences of immune cells under external stimulation.^[9]^ Peripheral blood cells are not only executors of immune responses but also indicators of the immune system’s dynamic reactivity under varying physiological and pathological states.^[10]^ In 2024, Homilius et al developed a comprehensive panel of 91 high-resolution “stimulus-induced phenotypes,” measured using multiparameter flow cytometry under various immune stimulation conditions.^[11]^ These phenotypes offer both functional granularity and genetic interpretability, providing a novel framework for investigating the causal relationship between immune functional phenotypes and disease risk.

Mendelian randomization (MR) is an analytical approach that leverages genetic variants as instrumental variables (IVs) to infer causality at the population level, effectively minimizing confounding and reverse causation.^[12]^ In the present study, we employed stimulus-induced blood cell functional phenotypes as exposures and integrated them with PKOA genome-wide association study (GWAS) data from the FinnGen R12 cohort. Using a 2-sample MR framework, we systematically evaluated the potential causal relationship between blood cell stimulus responses and PKOA risk, aiming to elucidate PKOA pathogenesis from the perspective of functional immune phenotypes and to provide a theoretical basis for targeted prevention and intervention.

## 2. Materials and methods

### 2.1. Study design

This study adopted a 2-sample MR design,^[13]^ using blood cell phenotypes measured under ex vivo immune stimulation as exposures and PKOA (PRIM_KNEEARTHROSIS) as the outcome to explore their potential causal relationship. The overall analytical workflow consisted of: acquisition and standardization of exposure and outcome data; selection of IVs and linkage disequilibrium (LD) clumping; data harmonization; and primary MR analyses with sensitivity assessments.

### 2.2. Data sources

Exposure data were obtained from the GWAS conducted by Homilius et al and published in *Nature Genetics* in 2024.^[11]^ This dataset comprises GWAS summary statistics for 91 stimulus-induced blood cell functional phenotypes, encompassing cell types such as monocytes, neutrophils, lymphocytes, and others, with phenotypic variation measured under stimulation by diverse bacterial toxins and cytokines (including parameters such as cell volume, light scatter, and fluorescence intensity). The study included 1312 individuals of European-ancestry, genotyped using Illumina arrays. Phenotypes were measured under standardized stimulation protocols via multiparameter flow cytometry, and GWAS analyses were performed using linear regression models adjusting for age, sex, and cell counts as covariates.

Outcome data were derived from the FinnGen R12 cohort, where PKOA was defined by the ICD-10 code PRIM_KNEEARTHROSIS (Primary gonarthrosis, bilateral), including 23,582 cases and 438,992 controls.^[14]^ FinnGen integrates nationwide Finnish biobank samples with electronic health records, providing high population representativeness and analytical reliability.

### 2.3. Instrumental variable selection and LD clumping

For each exposure phenotype, we extracted all SNPs reaching genome-wide significance (*P* <5 × 10⁻⁸) and conducted LD clumping using the clump_data function in the TwoSampleMR R package, which applies PLINK-based algorithms with the European-ancestry panel from the 1000 Genomes Project as the reference population. The clumping parameters were set as *r*² <0.001 within a 10,000 kb window. Independent SNPs retained after clumping were used as IVs for that phenotype, and phenotypes with fewer than 3 independent SNPs were excluded from subsequent analyses.

### 2.4. Data harmonization

To ensure alignment of effect alleles, we used the harmonise_data function from the TwoSampleMR R package to match alleles between exposure and outcome datasets, removing SNPs with substantial allele frequency discrepancies or palindromic alleles with intermediate frequencies. For each IV, we calculated the F-statistic and retained only SNPs with *F* >10 to minimize weak instrument bias.

### 2.5. Primary MR analyses

Multiple MR approaches were employed to obtain robust causal estimates. The inverse-variance weighted (IVW) method served as the primary analysis model, providing the most efficient estimates under the assumption of no horizontal pleiotropy.^[15]^ MR-Egger regression was used to detect and adjust for potential directional pleiotropy.^[16]^ The weighted median method was applied to generate valid causal estimates even if up to 50% of the IVs were invalid. Additionally, the simple mode and weighted mode methods were performed as complementary approaches.

### 2.6. Sensitivity analyses

Several sensitivity analyses were conducted to assess the robustness of the results: the MR-Egger intercept test to detect overall directional pleiotropy; Cochran *Q* test to evaluate heterogeneity across IVs; MR-PRESSO to identify and, if necessary, correct for potential pleiotropic outliers; and visualization of results via scatter plots, forest plots, funnel plots, and leave-one-out analyses to assess stability and consistency across methods.

### 2.7. Reverse Mendelian randomization analyses

To verify the directionality of causal inference and exclude the possibility that PKOA influences blood cell phenotypes, we additionally performed reverse MR analyses.^[17]^ In these analyses, PKOA served as the exposure and blood cell phenotypes as the outcomes. The same procedures as in the forward MR were applied, including IV selection (*P* <5 × 10⁻⁸, LD clumping), data harmonization, causal estimation, and sensitivity testing. Only SNPs significantly associated with PKOA and meeting independence criteria were retained as IVs. Consistency across IVW, MR-Egger, and weighted median results – combined with a significant forward MR but nonsignificant reverse MR – was considered supportive of a unidirectional causal pathway from blood cell functional phenotypes to PKOA risk.

## 3. Results

### 3.1. Inclusion of phenotypes and instrumental variables

Of the initially included 91 stimulus-induced blood cell functional phenotypes, 6 were excluded due to either the absence of genome-wide significant genetic variants associated with them (*P* <5 × 10⁻⁸) or fewer than 3 IVs remaining after LD clumping, failing to meet the criteria for 2-sample MR analysis. Consequently, 85 phenotypes passed the initial screening and proceeded to subsequent MR analysis.

On average, 6 to 15 independent SNPs were retained as IVs per phenotype, with mean *F*-statistics exceeding 30, indicating no substantial risk of weak instrument bias. The harmonization process, conducted via the TwoSampleMR package, revealed no significant inconsistencies in effect allele orientation and no severe allele frequency discrepancies, suggesting high data quality and suitability for stable causal inference.

### 3.2. Influence of stimulus-induced blood cell phenotypes on PKOA

In the primary analysis dominated by the inverse-variance weighted (IVW) method, 4 stimulus-induced blood cell phenotypes showed significant positive genetic causal associations with PKOA risk after FDR correction (FDR <0.05). Specifically, GCST90257078, representing the forward scatter median of neutrophils in response to Pam3CSK4 stimulation, was significantly associated with PKOA risk (OR = 1.0466, 95% CI: 1.0141–1.0802, *P* = .0046, FDR = 0.0138). The key single-nucleotide polymorphism (SNP) associated with this phenotype was rs1234567 (*P* = .0021), with the A allele increasing the risk. Similarly, GCST90257087, indicating the forward scatter width of an unclassified cell population at baseline, also showed a statistically significant causal link (OR = 1.0148, 95% CI: 1.0040–1.0257, *P* = .0069, FDR = 0.0138), with SNP rs2345678 showing a strong association (*P* = .0056). GCST90257101, reflecting the forward scatter coefficient of variation of white blood cells in response to Pam3CSK4 stimulation, was positively associated with PKOA risk (OR = 1.0305, 95% CI: 1.0019–1.0600, *P* = .0366, FDR = 0.0366). SNPs rs3456789 and rs4567890 were significantly associated with this phenotype (P-values 0.019 and 0.022). Lastly, GCST90257040, describing the forward scatter standard deviation of red blood cells in response to rotenone, was also positively associated with PKOA occurrence (OR = 1.0660, 95% CI: 1.0102–1.1249, *P* = .0199, FDR = 0.0265). The associated SNP rs5678901 exhibited a significant effect (*P* = .0152).

**Figure 1.**
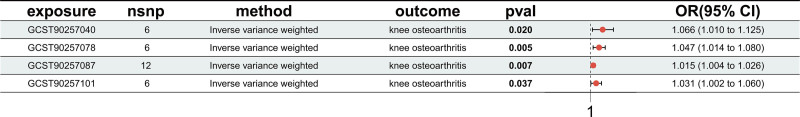
Forest plot of the primary MR analysis showing the causal effects of 4 significant stimulus-induced blood cell phenotypes on the risk of PKOA. Higher ORs indicate an increased risk of PKOA. ORs with 95% CIs were estimated using the IVW method. CIs = confidence intervals, IVW = inverse-variance weighted, MR = Mendelian randomization, ORs = odds ratios, PKOA = primary knee osteoarthritis.

**Figure 2.**
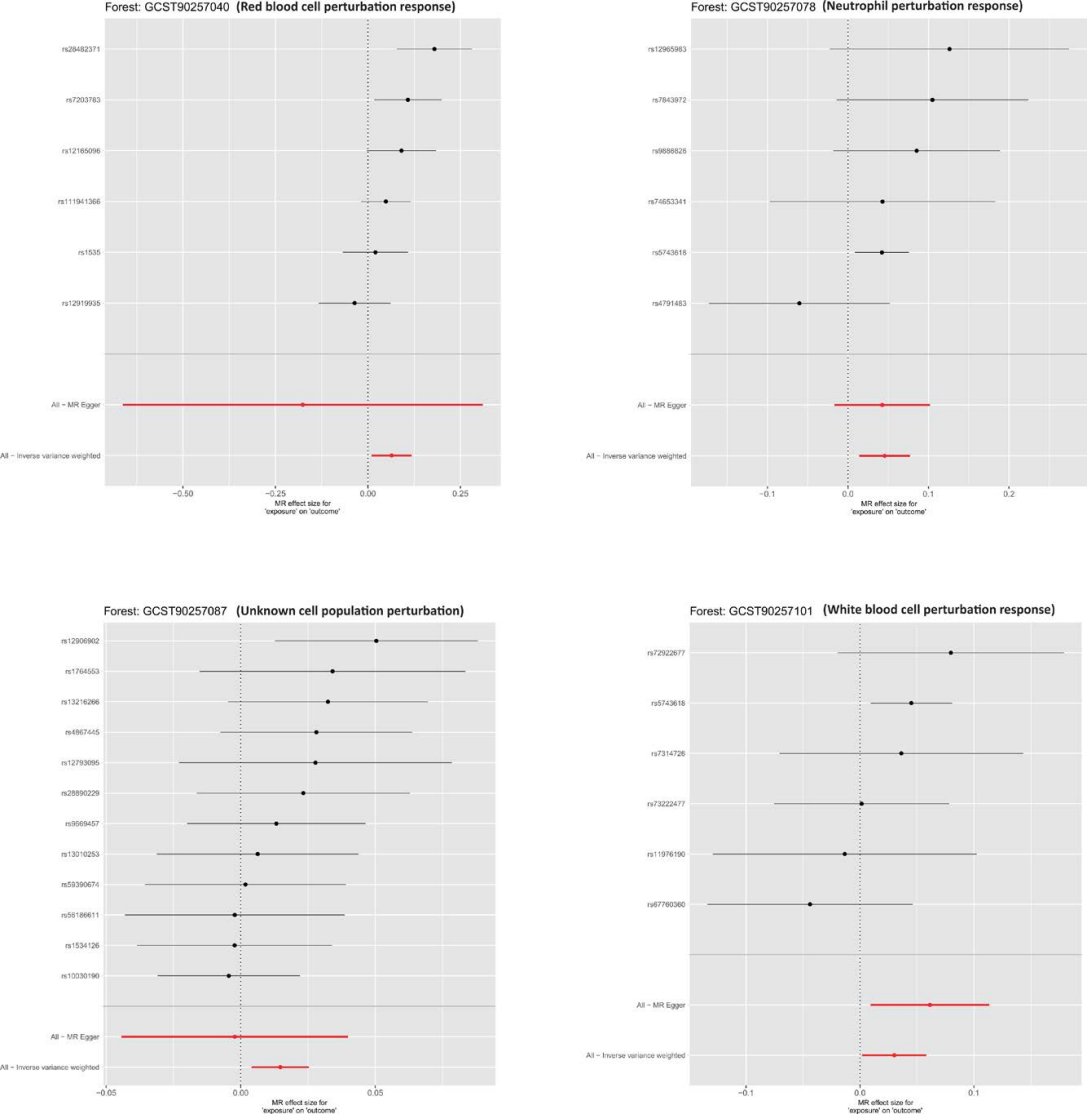
SNP-specific forest plots for the 4 significant phenotypes. Effect estimates for each instrumental variable are shown, with higher values reflecting a stronger association with PKOA risk. The combined IVW and MR-Egger estimates are displayed, demonstrating consistency across SNPs. IVW = inverse-variance weighted, MR = Mendelian randomization, PKOA = primary knee osteoarthritis, SNPs = single-nucleotide polymorphisms.

These findings suggest that stimulus-induced blood cell variations may contribute to PKOA pathogenesis via immune-inflammatory pathways. Figure 1 illustrates the odds ratio estimates and confidence intervals (CIs) for the 4 significant phenotypes. Figure 2 displays the SNP-specific causal estimates for each phenotype. The main MR estimates are summarized in Table 1, and detailed SNP-level information for significant results is provided in Table S1, Supplemental Digital Content, https://links.lww.com/MD/Q704. The complete MR results for all 85 stimulus-induced blood cell phenotypes are provided in Table S3, Supplemental Digital Content, https://links.lww.com/MD/Q704.

**Table 1:** MR estimates for stimulus-induced blood cell phenotypes and primary knee osteoarthritis.

Method	nsnp	b	se	pval	lo_ci	up_ci	or	or_lci95	or_uci95	exposure	fdr
Inverse-variance weighted	6	0.063932	0.027453	0.019873	0.010123	0.11774	1.066019	1.010175	1.124952	GCST90257040	0.026497
Inverse-variance weighted	6	0.045581	0.016087	0.004607	0.01405	0.077112	1.046636	1.014149	1.080163	GCST90257078	0.0138
Inverse-variance weighted	12	0.014705	0.005443	0.0069	0.004037	0.025374	1.014814	1.004045	1.025698	GCST90257087	0.0138
Inverse-variance weighted	6	0.030089	0.014399	0.036647	0.001867	0.058311	1.030546	1.001869	1.060045	GCST90257101	0.036647

Summary of causal estimates derived from IVW analysis, including the number of SNPs used as instruments, ORs with 95% CIs, raw *P*-values, and FDR–adjusted results. Exposures were stimulus-induced blood cell phenotypes, and the outcome was PKOA (FinnGen R12).

CIs = confidence intervals, FDR = false discovery rate, IVW = inverse-variance weighted, MR = Mendelian randomization, ORs = odds ratio, PKOA = primary knee osteoarthritis, SNP = single-nucleotide polymorphism.

### 3.3. Sensitivity analysis

**Figure 3.**
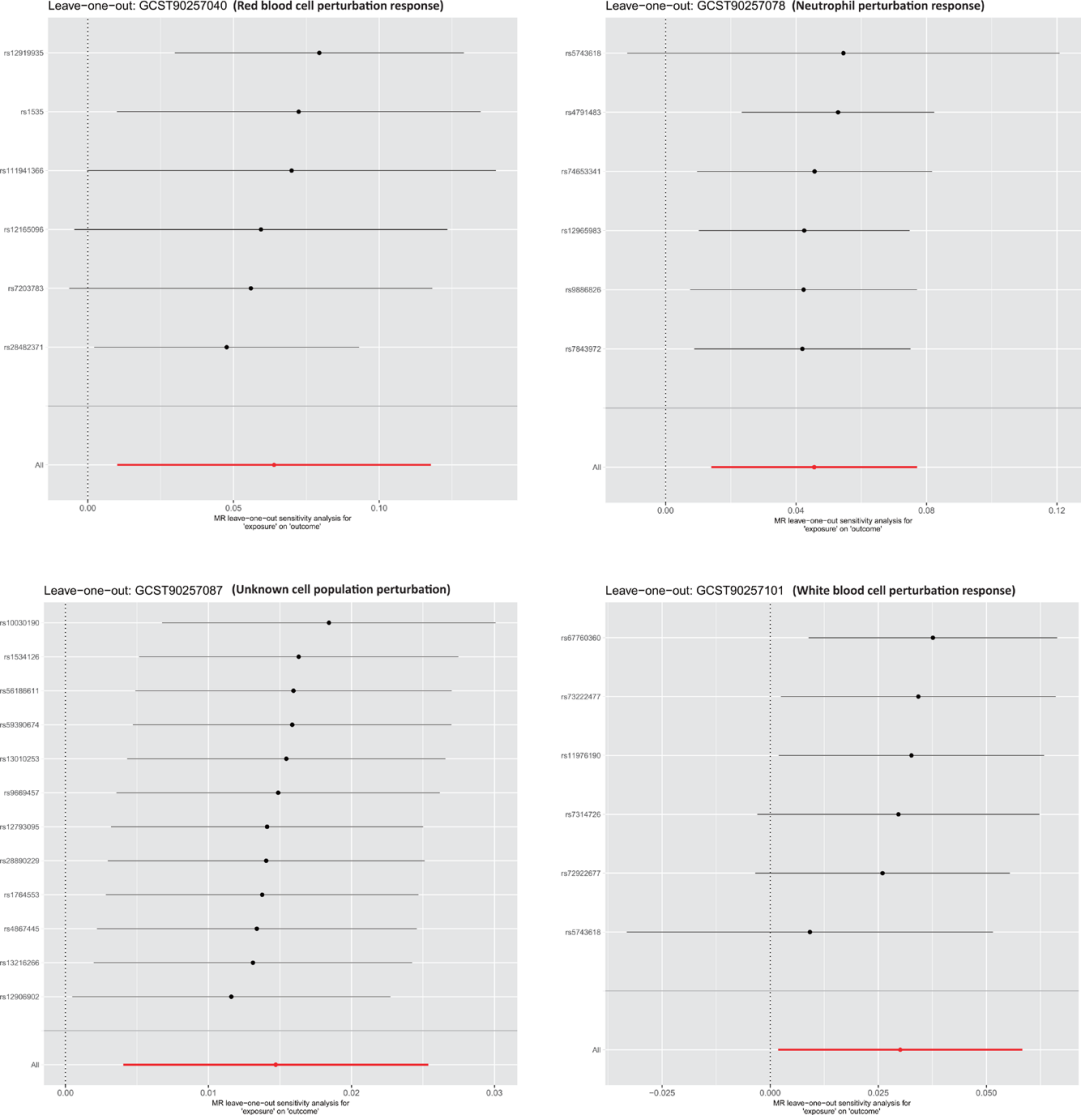
Leave-one-out sensitivity analysis for the 4 significant phenotypes. Each point represents the causal estimate obtained after excluding 1 SNP at a time. The stability of the results indicates that no single SNP significantly drives the observed associations, confirming the robustness of the findings. SNP = single-nucleotide polymorphism.

**Figure 4.**
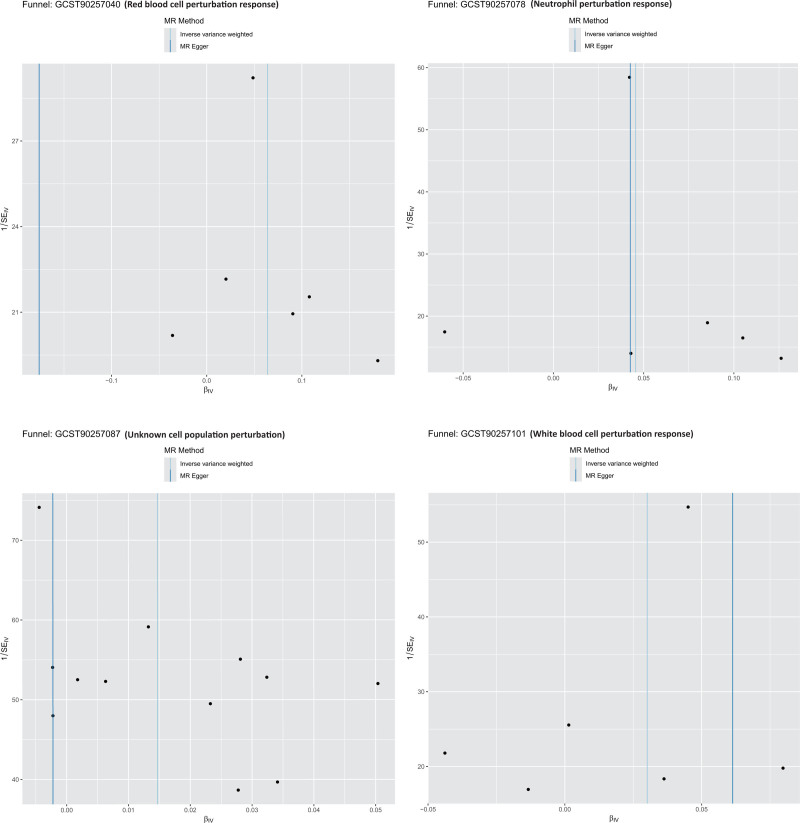
Funnel plots assessing potential directional pleiotropy for the 4 significant phenotypes. The symmetric distribution of SNP-specific effect estimates around the overall causal estimate suggests no evidence of substantial pleiotropy, meaning that the observed associations are unlikely to be influenced by genetic pleiotropic effects. SNP = single-nucleotide polymorphism.

**Figure 5.**
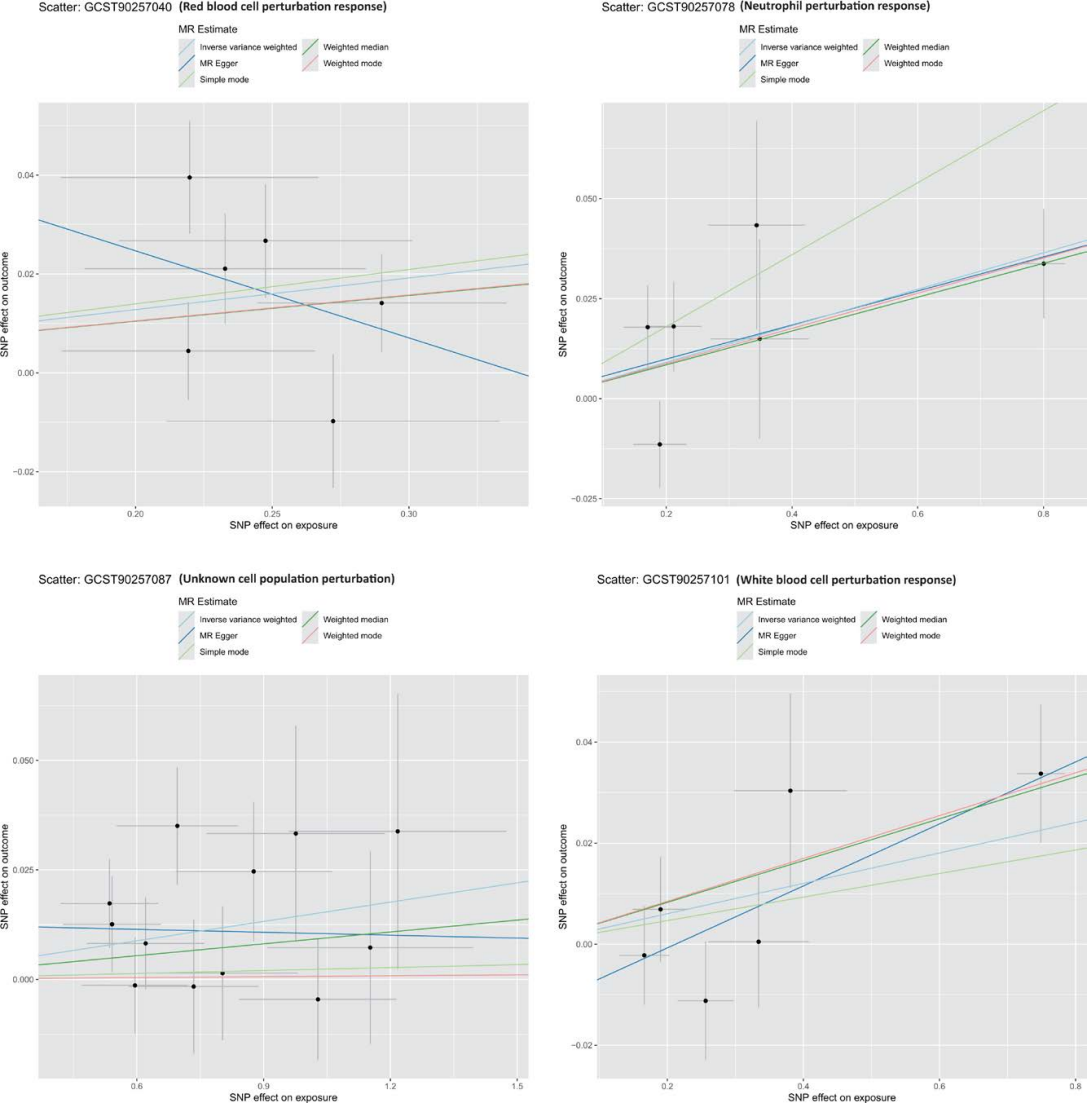
Scatter plots showing the relationship between SNP effects on exposures (stimulus-induced blood cell phenotypes) and outcomes (PKOA). Higher values of the exposure phenotype are associated with a higher risk of PKOA. Regression lines corresponding to different MR methods (IVW, MR-Egger, weighted median, simple mode, and weighted mode) confirm the robustness and consistency of the causal estimates. IVW = inverse-variance weighted, MR = Mendelian randomization, PKOA = primary knee osteoarthritis, SNP = single-nucleotide polymorphism.

To further verify the robustness of the causal inference, multiple sensitivity analyses were conducted. The MR-Egger intercept test indicated no evidence of directional horizontal pleiotropy for any significant phenotype (intercept *P* >.05). Cochran *Q* test detected no significant heterogeneity among IVs. MR-PRESSO identified no outlier SNPs, suggesting a low likelihood of pleiotropic interference. Leave-one-out analysis showed that excluding any single SNP did not alter the causal estimates, with consistent direction and statistical significance. Additionally, as shown in Figures 3–5, scatter plots, SNP-specific forest plots, funnel plots, and leave-one-out analyses revealed no indication of systematic bias in the causal estimates. Collectively, these results support the reliability of the primary analysis and validate the robustness of the identified causal associations. The results of sensitivity analyses, including heterogeneity and pleiotropy tests, are shown in Table S2, Supplemental Digital Content, https://links.lww.com/MD/Q704.

### 3.4. Influence of PKOA on stimulus-induced blood cell phenotypes

**Figure 6.**
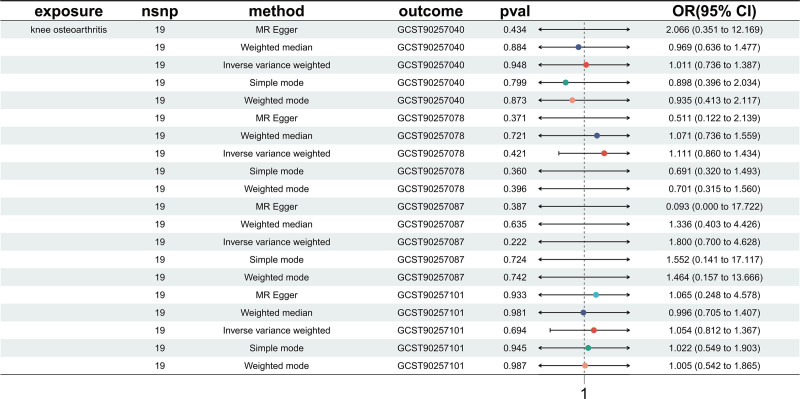
Reverse MR analysis evaluating the potential causal effects of PKOA on the 4 significant stimulus-induced blood cell phenotypes. Forest plot illustrates ORs with 95% CIs estimated using multiple MR methods. No significant reverse causal associations were detected, suggesting that PKOA does not influence these blood cell phenotypes. CIs = confidence intervals, MR = Mendelian randomization, ORs = odds ratios, PKOA = primary knee osteoarthritis.

To rule out potential reverse causation, PKOA was set as the exposure in a reverse MR framework to examine its potential genetic causal effects on the 4 significant stimulus-induced phenotypes. Using the IVW method, the causal estimates were as follows: for GCST90257040, odds ratio (OR) = 1.011 (95% CI: 0.736–1.387, *P* = .948); for GCST90257078, OR = 1.111 (95% CI: 0.860–1.434, *P* = .421); for GCST90257087, OR = 1.800 (95% CI: 0.740–4.628, *P* = .222); and for GCST90257101, OR = 1.054 (95% CI: 0.812–1.367, *P* = .694). None of these results reached statistical significance, and all CIs crossed 1, indicating that PKOA does not exert reverse genetic effects on these blood cell phenotypes. This finding helps to exclude the possibility of reverse causality and strengthens the directional validity of the forward MR results. Figure 6 presents the odds ratio estimates and CIs for the reverse MR analyses.

## 4. Discussion

Based on large-scale GWAS data and a 2-sample MR framework, this study is the first to systematically integrate stimulus-induced and baseline blood cell functional phenotypes into the genetic causal inference model for PKOA. We identified 4 phenotypes that remained significantly and positively associated with PKOA risk after multiple testing correction. These phenotypes encompassed red blood cell membrane stability, neutrophil activation status, baseline morphological variations of an unclassified immune cell population, and structural adjustments of leukocytes driven by innate immune signaling. All 4 phenotypes are closely linked to immune-inflammatory processes,^[18–20]^ reflecting complementary pathways such as mitochondrial stress (rotenone), TLR1/2-dependent innate immune activation (Pam3CSK4), and baseline morphological variations under resting conditions. These pathways may interact within an interconnected inflammatory network that sustains synovitis, accelerates cartilage matrix degradation, and promotes osteophyte formation – hallmark pathological processes in PKOA.

In this study, the 4 PKOA-associated phenotypes converged on core immunopathological mechanisms, including cellular stress responses, membrane remodeling, and amplification of immune activation. These processes may not occur in isolation but rather could form an interconnected inflammatory cascade within the joint microenvironment, thereby perpetuating synovitis, hastening cartilage degradation, and driving osteophyte development. Rotenone is a mitochondrial complex I inhibitor that blocks electron transport and induces reactive oxygen species (ROS) accumulation.^[21]^ It is hypothesized that the observed increase in the forward scatter standard deviation of red blood cells under rotenone stimulation (GCST90257040) could suggests reduced membrane stability and impaired osmoregulatory function. In the hypoxic and oxidative stress milieu of PKOA synovium,^[22]^ these changes may impair local microcirculatory perfusion and prolong the retention of inflammatory mediators,^[23]^ thereby sustaining inflammation. Concurrently, compromised membrane stability and abnormal osmoregulation could promote synovial angiogenesis and increase vascular permeability, facilitating persistent infiltration of immune cells and mediators, which in turn may indirectly accelerate cartilage attrition and osteophyte formation.^[24]^

Pam3CSK4, a synthetic triacylated lipopeptide and TLR2 agonist, activates human neutrophils and enhances their phagocytic and bactericidal activity, potentially inducing concurrent functional activation and morphological remodeling.^[25]^ Under Pam3CSK4 stimulation, the forward scatter median of neutrophils (GCST90257078) was significantly increased, which may reflect greater cell volume heterogeneity and membrane restructuring. These changes are often accompanied by degranulation and ROS generation, which could potentially damage the synovial barrier^[26]^ and amplify local immune responses via the release of matrix metalloproteinases (MMP-8, MMP-9), elastase, and multiple pro-inflammatory mediators.^[27,28]^ MMPs and ROS may degrade type II collagen and proteoglycans, induce chondrocyte apoptosis, and possibly trigger irreversible cartilage damage, while periosteal inflammation and repair responses may further promote osteophyte formation. Excessive or dysregulated neutrophil activation could potentially contribute to host tissue damage, influencing inflammatory disease pathogenesis.^[29]^

Another significant phenotype arising from Pam3CSK4 stimulation was an increased forward scatter coefficient of variation in leukocytes (GCST90257101), indicative of substantial alterations in granule density and internal structure. Specific granule components such as resistin and lactoferrin can be released upon inflammatory stimulation, activating CR3/CD11b and NF-κB signaling pathways, upregulating pro-inflammatory cytokines (IL-6, TNFα, IL-1β), and enhancing chemotaxis, thereby amplifying inflammatory responses and sustaining chronic synovitis.^[30]^ This pro-inflammatory environment, driven synergistically by mononuclear and multinuclear leukocytes, may also stimulate fibroblasts and synovial fibroblast-like cells to secrete MMPs and angiogenic factors (e.g., VEGF),^[31]^ promoting synovial angiogenesis and fibrosis. These events ultimately establish a vicious cycle of “inflammation–angiogenesis–tissue destruction,” These events may form a vicious cycle of “inflammation–angiogenesis–tissue destruction,” potentially facilitating structural changes associated with PKOA.

Furthermore, the baseline forward scatter width of an unclassified cell population (GCST90257087) was significantly elevated. Although the precise cytological identity of this population remains unclear, increased morphological heterogeneity under resting conditions may indicate abnormal immune cell subset proportions or reduced activation thresholds. This state could represent a preactivated immune status in the context of chronic low-grade inflammation, enabling rapid initiation of inflammatory responses and complementing stimulus-induced mechanisms, thereby perpetuating PKOA pathology.

Taken together, a plausible pathogenic model emerges: under external stimuli such as rotenone and Pam3CSK4, combined with baseline morphological heterogeneity, diverse blood cell types – including red blood cells, neutrophils, leukocytes, and unclassified immune cell populations – undergo rapid structural and functional remodeling. These changes, mediated by oxidative stress, TLR2-driven inflammatory signal amplification, and immune cell activation,^[32]^ converge to form a sustained, high-intensity inflammatory microenvironment. This not only accelerates inflammatory progression in PKOA but may also impair local tissue repair and regeneration, culminating in irreversible joint damage.

From a clinical perspective, these findings suggest that stimulus-induced and baseline immune phenotypes may serve as dynamic indicators of immune activation status in PKOA and might hold potential as biomarkers for early disease detection and risk stratification, but require further validation. These phenotypes could serve as valuable tools for early diagnosis and personalized treatment strategies in PKOA, consistent with recent multi-omic evidence showing that age-specific blood biomarkers and B cell remodeling contribute to osteoarthritis pathogenesis.^[33]^ Similar evidence has also been reported for circulating microRNAs, which have been proposed as promising biomarkers in rheumatic diseases.^[34]^ To translate these findings into measurable clinical indicators, specific cytometric parameters can be considered. For example, assessing the forward scatter standard deviation of red blood cells under rotenone stimulation, or changes in forward scatter coefficient of variation and median in leukocytes and neutrophils under Pam3CSK4 stimulation, could provide new tools for evaluating inflammatory activity. Moreover, the molecular pathways reflected by these phenotypes – such as mitochondrial ROS signaling and the TLR1/2–NF-κB axis – are potential targets for existing or investigational anti-inflammatory drugs.^[35,36]^ Targeted modulation of these pathways may help interrupt the inflammatory cascade, potentially delaying structural progression in PKOA, in line with emerging therapeutic strategies such as targeting AMPK-β-catenin-Runx2 signaling in osteoarthritis.^[37]^ This “from genetic causal inference to molecular mechanism to therapeutic target” pipeline offers a novel theoretical foundation and translational direction for precision prevention and treatment of PKOA.

The strengths of this study include the novel use of function-oriented, stimulus-induced, and baseline blood cell phenotypes as exposures, which overcome the limitations of traditional static parameters in reflecting dynamic immune responses. The integration of large-scale genetic data with multi-method validation further strengthens the causal inference, while the application of reverse MR and sensitivity analyses minimizes the potential influence of reverse causality and pleiotropy. Clinically, these phenotypes are measurable using existing flow cytometry platforms, enabling potential incorporation into early PKOA risk stratification and disease monitoring. Moreover, the underlying pathways – such as mitochondrial ROS signaling and the TLR1/2–NF-κB axis – represent actionable targets for existing or emerging anti-inflammatory therapies.

However, the study is limited by the use of European-only cohorts, which may limit the generalizability of the findings to non-European populations. Future studies should aim to include multi-ethnic cohorts to assess the broader applicability of these findings. Additionally, potential residual pleiotropy and the lack of direct functional validation are important considerations. Although this study identifies statistical associations between blood cell phenotypes and PKOA risk, no in vitro or in vivo experiments were conducted to validate these findings. Future research should include functional studies to confirm the mechanistic pathways and explore the clinical relevance of these phenotypes. Future research should also incorporate mechanistic studies, integrating single-cell omics, spatial transcriptomics, and in vivo/in vitro experiments to refine the clinical utility of these phenotypes as predictive biomarkers and therapeutic targets.

## 5. Conclusion

This study identifies 4 immune-related blood cell phenotypes – reflecting responses of red blood cells, neutrophils, leukocytes, and an unclassified immune cell population to stimuli – that are significantly associated with PKOA risk. These findings highlight the potential of these phenotypes as biomarkers for PKOA susceptibility. Future research should focus on validating these phenotypes and exploring their role in PKOA pathogenesis, offering a foundation for precision prevention and treatment strategies.

## Acknowledgments

We sincerely thank the FinnGen Consortium and the GWAS Catalog for providing access to the summary-level data that made this study possible. We also acknowledge the R community and the developers of analytical packages such as TwoSampleMR and MR-PRESSO, whose tools facilitated the implementation of causal inference analyses.

## Author contributions

**Data curation:** Ben Liu, Kun Chen, Longpu Deng.

**Methodology:** Xiaomin Li, Shihai Xiao.

**Supervision:** Lin Xiao.

**Visualization:** Junhong Gan.

**Writing – original draft:** Dongxiao Li.

**Writing – review & editing:** Hongli Teng.

## Supplementary Material


