# Supplementary material for: Causal effects of stimulus-induced blood cell phenotypes on the risk of primary knee osteoarthritis: A two-sample Mendelian randomization study
Source: Medicine (Baltimore). 2025 Nov 14;104(46):e45999. doi: 10.1097/MD.0000000000045999 (PMC12622675; doi:10.1097/MD.0000000000045999)
Supplement: Supplementary file 1 [file medi-104-e45999-s001.pdf]

Supplementary Table 1:SNP for significant MR analysis

exposure	outcome	SNP	b	se	p	lo_ci	up_ci	or	or_lci95	or_uci95	id
Red blood cell perturbation response (forward scatter standard deviation of RBC in response to rotenone perturbation measured by reticulocyte dye)	Primary knee osteoarthritis	rs111941366	0.0486526739664065	0.0342328722729404	0.155250849054765	-0.018443756	0.11574910362137	1.04985564521074	0.981725289501949	1.12271415187849	GCST90257040
Red blood cell perturbation response (forward scatter standard deviation of RBC in response to rotenone perturbation measured by reticulocyte dye)	Primary knee osteoarthritis	rs12165096	0.0904846394095484	0.0477439199900331	0.0580649053794596	-0.003093444	0.184062722590013	1.09470469220206	0.996911335996348	1.20209121900645	GCST90257040
Red blood cell perturbation response (forward scatter standard deviation of RBC in response to rotenone perturbation measured by reticulocyte dye)	Primary knee osteoarthritis	rs12919935	-0.0359304	0.0495363484456196	0.468246590989145	-0.133021686	0.0611607998677905	0.964707393219019	0.875446106944464	1.06306984193428	GCST90257040
Red blood cell perturbation response (forward scatter standard deviation of RBC in response to rotenone perturbation measured by reticulocyte dye)	Primary knee osteoarthritis	rs1535	0.0202201825659545	0.0451259358556981	0.654093011013432	-0.068226652	0.108667016843123	1.02042599530727	0.934048745837291	1.1147910818781	GCST90257040
Red blood cell perturbation response (forward scatter standard deviation of RBC in response to rotenone perturbation measured by reticulocyte dye)	Primary knee osteoarthritis	rs28482371	0.17978862794697	0.0518057160535391	0.000519606464578006	0.0782494244820338	0.281327831411907	1.19696433157269	1.08139235076174	1.32488787260981	GCST90257040
Red blood cell perturbation response (forward scatter standard deviation of RBC in response to rotenone perturbation measured by reticulocyte dye)	Primary knee osteoarthritis	rs7203783	0.107863770586501	0.0464264218167423	0.0201615793959261	0.0168679838256858	0.198859557347316	1.11389598965254	1.01701105155422	1.22001061234078	GCST90257040
Red blood cell perturbation response (forward scatter standard deviation of RBC in response to rotenone perturbation measured by reticulocyte dye)	Primary knee osteoarthritis	All - Inverse variance weighted	0.0639315942949385	0.0274532743276118	0.0198727351084882	0.0101231766128193	0.117740011977058	1.06601947445356	1.01017458930532	1.12495159940195	GCST90257040
Red blood cell perturbation response (forward scatter	Primary	All - MR	-	0.248036	0.516963	-	0.31007	0.83855	0.51569	1.3635	GCST

exposure	outcome	SNP	b	se	p	lo_ci	up_ci	or	or_lci95	or_uci95	id
standard deviation of RBC in response to rotenone perturbation measured by reticulocyte dye)	knee osteoarthritis	Egger	0.1760796	622136689	20177604	0.6622314	2159198808	1215072742	9318424623	2350134578	90257040
Neutrophil perturbation response (forward scatter median of neutrophil 1 in response to Pam3CSK4 perturbation measured by WDF dye)	Primary knee osteoarthritis	rs12965983	0.126174207591754	0.0757473023780978	0.0957681151520685	-0.022290505	0.274638920252826	1.13447978606069	0.97795609257833	1.31605538811777	GCST90257078
Neutrophil perturbation response (forward scatter median of neutrophil 1 in response to Pam3CSK5 perturbation measured by WDF dye)	Primary knee osteoarthritis	rs4791483	-0.0602294	0.0572883553952966	0.293103393495764	-0.1725146	0.0520557532433985	0.941548495610599	0.841546002229417	1.05343447326473	GCST90257078
Neutrophil perturbation response (forward scatter median of neutrophil 1 in response to Pam3CSK6 perturbation measured by WDF dye)	Primary knee osteoarthritis	rs5743618	0.0421673334449489	0.0171106718676206	0.0137247471641382	0.00863041658441264	0.0757042503054852	1.0430690044782	1.00866776599931	1.07864351848822	GCST90257078
Neutrophil perturbation response (forward scatter median of neutrophil 1 in response to Pam3CSK7 perturbation measured by WDF dye)	Primary knee osteoarthritis	rs74653341	0.0429389778291563	0.0714610267604866	0.547924798711063	-0.097124635	0.18300259027971	1.04387419343766	0.90744290028265	1.20081751853006	GCST90257078
Neutrophil perturbation response (forward scatter median of neutrophil 1 in response to Pam3CSK8 perturbation measured by WDF dye)	Primary knee osteoarthritis	rs7843972	0.10492595808594	0.0606768205392974	0.0837626918640765	-0.01400061	0.223852526342963	1.11062837426064	0.986096942574839	1.25088653301369	GCST90257078
Neutrophil perturbation response (forward scatter median of neutrophil 1 in response to Pam3CSK9 perturbation measured by WDF dye)	Primary knee osteoarthritis	rs9886826	0.0853629622632598	0.0528356869598073	0.106174164407266	-0.018194984	0.188920908704482	1.08911230163232	0.981969545165873	1.20794541073724	GCST90257078
Neutrophil perturbation response (forward scatter median of neutrophil 1 in response to Pam3CSK10 perturbation measured by WDF dye)	Primary knee osteoarthritis	All - Inverse variance weighted	0.0455807481237503	0.0160873483215878	0.0046065968747371	0.0140495454134383	0.0771119508340624	1.04663551505596	1.01414870411061	1.08016299477221	GCST90257078

exposure	outcome	SNP	b	se	p	lo_ci	up_ci	or	or_lci95	or_uci95	id
Neutrophil perturbation response (forward scatter median of neutrophil 1 in response to Pam3CSK11 perturbation measured by WDF dye)	Primary knee osteoarthritis	All - MR Egger	0.0425734455663548	0.0302378261895166	0.231909780820995	-0.016692694	0.101839584897807	1.04349269347118	0.983445857246388	1.10720584494254	GCST90257078
Unknown cell population perturbation response (forward scatter coefficient of variation of unknown cell population 1 at baseline measured by WNR dye)	Primary knee osteoarthritis	rs10030190	-0.0044317	0.0134905201750122	0.742528604461128	-0.030873145	0.0220096937287312	0.995578079791953	0.969598563354218	1.02225369387241	GCST90257087
Unknown cell population perturbation response (forward scatter coefficient of variation of unknown cell population 2 at baseline measured by WNR dye)	Primary knee osteoarthritis	rs12793095	0.027761019928697	0.0258691902015871	0.283212517588496	-0.022942593	0.0784646327238076	1.02814994771169	0.977318587221586	1.08162510035214	GCST90257087
Unknown cell population perturbation response (forward scatter coefficient of variation of unknown cell population 3 at baseline measured by WNR dye)	Primary knee osteoarthritis	rs12906902	0.050376863837457	0.0192259486993742	0.00878643657157811	0.0126940043866836	0.0880597232882304	1.05166735709909	1.01277491525889	1.09205334109708	GCST90257087
Unknown cell population perturbation response (forward scatter coefficient of variation of unknown cell population 4 at baseline measured by WNR dye)	Primary knee osteoarthritis	rs13010253	0.0063023249759171	0.0191217336214602	0.741710064503401	-0.031176273	0.0437809228739792	1.00632222641244	0.969304695844769	1.04475344823241	GCST90257087
Unknown cell population perturbation response (forward scatter coefficient of variation of unknown cell population 5 at baseline measured by WNR dye)	Primary knee osteoarthritis	rs13216266	0.0323953632587191	0.0189362242610801	0.0871251510288907	-0.004719636	0.069510362810436	1.03292580549883	0.995291483689402	1.07198316990559	GCST90257087
Unknown cell population perturbation response (forward scatter coefficient of variation of unknown cell population 6 at baseline measured by WNR dye)	Primary knee osteoarthritis	rs1534126	-0.002266	0.0185007773647503	0.902518838998231	-0.0385275	0.0339955473621647	0.997736589113421	0.962205243820631	1.03458000010781	GCST90257087
Unknown cell population perturbation response (forward scatter coefficient of variation of unknown cell population 7 at baseline measured by WNR dye)	Primary knee osteoarthri	rs1764553	0.0341133204257455	0.0252089287726523	0.17598416895507	-0.01529618	0.083522820820144	1.03470185293929	0.984820212383683	1.08711002375213	GCST90257087

exposure	outcome	SNP	b	se	p	lo_ci	up_ci	or	or_lci95	or_uci95	id
	tis										
Unknown cell population perturbation response (forward scatter coefficient of variation of unknown cell population 8 at baseline measured by WNR dye)	Primary knee osteoarthritis	rs28890229	0.0232563853863563	0.0202056256062076	0.249738133782904	-0.016346641	0.0628594115745231	1.02352892376861	0.98378624049144	1.06487711930958	GCST90257087
Unknown cell population perturbation response (forward scatter coefficient of variation of unknown cell population 9 at baseline measured by WNR dye)	Primary knee osteoarthritis	rs4867445	0.0281193954246129	0.0181577974617691	0.121474855484299	-0.007469888	0.0637086784496803	1.02851847749116	0.99255794267048	1.06578186830542	GCST90257087
Unknown cell population perturbation response (forward scatter coefficient of variation of unknown cell population 10 at baseline measured by WNR dye)	Primary knee osteoarthritis	rs56186611	-0.0022225	0.0208369129389981	0.915058058754434	-0.043062832	0.0386178669407816	0.997779985465782	0.957851204680021	1.03937322888128	GCST90257087
Unknown cell population perturbation response (forward scatter coefficient of variation of unknown cell population 11 at baseline measured by WNR dye)	Primary knee osteoarthritis	rs59390674	0.00177142280590084	0.0190435736876262	0.925888107036347	-0.035553982	0.0390968272336482	1.00177299270213	0.965070636745692	1.03987116662407	GCST90257087
Unknown cell population perturbation response (forward scatter coefficient of variation of unknown cell population 12 at baseline measured by WNR dye)	Primary knee osteoarthritis	rs9669457	0.0132314265552652	0.0169111226393512	0.433973829300826	-0.019914374	0.0463772269283935	1.01331934923247	0.98028260757032	1.04746947012958	GCST90257087
Unknown cell population perturbation response (forward scatter coefficient of variation of unknown cell population 13 at baseline measured by WNR dye)	Primary knee osteoarthritis	All - Inverse variance weighted	0.0147051339171546	0.00544307692185642	0.00690022533638774	0.00403670315031598	0.0253735646839931	1.01481378632839	1.00404486161054	1.02569821359399	GCST90257087
Unknown cell population perturbation response (forward scatter coefficient of variation of unknown cell population 14 at baseline measured by WNR dye)	Primary knee osteoarthritis	All - MR Egger	-0.0022215	0.0214663669110363	0.919623230942896	-0.044295548	0.0398526101711452	0.9977809966616	0.956671173320098	1.04065738057512	GCST90257087
White blood cell perturbation response (forward scatter coefficient of variation of WBC in response to	Primary knee	rs11976190	-0.013357	0.0590337486143	0.8209929493436	-0.129063	0.102348696322	0.98673136390	0.87891806612	1.1077696796	GCST90257

exposure	outcome	SNP	b	se	p	lo_ci	up_ci	or	or_lci95	or_uci95	id
Pam3CSK4 perturbation measured by WNR dye)	osteoarthritis		5	911	48	598	714	0095	0931	0308	101
White blood cell perturbation response (forward scatter coefficient of variation of WBC in response to Pam3CSK5 perturbation measured by WNR dye)	Primary knee osteoarthritis	rs5743618	0.0450617926820801	0.0182851863126806	0.0137247471641382	0.00922282750922618	0.0809007578549341	1.0460924987728	1.00926548883468	1.08426328661275	GCST90257101
White blood cell perturbation response (forward scatter coefficient of variation of WBC in response to Pam3CSK6 perturbation measured by WNR dye)	Primary knee osteoarthritis	rs67760360	-0.0438267	0.0458743993785935	0.339393931565996	-0.133740547	0.046087098398473	0.957119788626756	0.874817008913921	1.04716561343295	GCST90257101
White blood cell perturbation response (forward scatter coefficient of variation of WBC in response to Pam3CSK7 perturbation measured by WNR dye)	Primary knee osteoarthritis	rs72922677	0.0797404841110846	0.050538907691217	0.114610674632434	-0.019315775	0.17879674318587	1.08300597394436	0.980869579278736	1.19577766950592	GCST90257101
White blood cell perturbation response (forward scatter coefficient of variation of WBC in response to Pam3CSK8 perturbation measured by WNR dye)	Primary knee osteoarthritis	rs7314726	0.0362539816252929	0.0544833758917524	0.505785961284596	-0.070533435	0.143041398373128	1.03691917146612	0.931896580928528	1.15377756519149	GCST90257101
White blood cell perturbation response (forward scatter coefficient of variation of WBC in response to Pam3CSK9 perturbation measured by WNR dye)	Primary knee osteoarthritis	rs73222477	0.00143318128213877	0.0391379176169866	0.970789000116255	-0.075277137	0.0781434998114326	1.00143420877724	0.927486409677089	1.08127781069962	GCST90257101
White blood cell perturbation response (forward scatter coefficient of variation of WBC in response to Pam3CSK10 perturbation measured by WNR dye)	Primary knee osteoarthritis	All - Inverse variance weighted	0.0300890984891457	0.0143989183999893	0.0366467627384539	0.00186721842516668	0.0583109785531247	1.0305463499859	1.00186896276301	1.06004459559297	GCST90257101
White blood cell perturbation response (forward scatter coefficient of variation of WBC in response to Pam3CSK11 perturbation measured by WNR dye)	Primary knee osteoarthritis	All - MR Egger	0.0613563134276172	0.026602088071037	0.0823531469740355	0.00921622080838466	0.11349640604685	1.06327770682296	1.00925882094155	1.12018786298245	GCST90257101

Supplementary Table 2:Sensitivity analyses for the causal associations between stimulus-induced blood cell phenotypes and Primary knee osteoarthritis

id.exposure	id.outcome	egger_intercept	se	pval	method	Q	Q_df	Q_pval	MR-PRESSO Global P	Causal_Estimate_Raw	P-value_Raw	Sd_Raw	id
Y0nGkJ	IZ5OXY	0.059889268	0.061507546	0.385345716	Inverse variance weighted	11.407608015965	5	0.0438711651601689	0.089	0.0639315942949385	0.0673166114892696	0.0274532743276118	GCST90257040
v206p9	2y1Wyl	0.001387854	0.011229155	0.907598462	Inverse variance weighted	6.10804105956848	5	0.295847589253937	0.511	0.0455807481237503	0.0365315388327336	0.0160873483215878	GCST90257078
8zKBTh	UTmTWQ	0.01276763	0.015662775	0.433967091	Inverse variance weighted	10.0632099045366	11	0.524710503756045	0.665	0.0144453051153433	0.0084192635623707	0.0046572302446549	GCST90257087
g2LqpK	XAjQYA	-0.013017118	0.009428494	0.239527001	Inverse variance weighted	5.32239184510059	5	0.377814350736979	0.42	0.0300890984891457	0.0909370721522552	0.0143989183999893	GCST90257101

Supplementary Table 3. Complete Mendelian randomization results

id.exposure	id.outcome	outcome	exposure	method	nsnp	b	se	pval	lo_ci	up_ci	or	or_lci95	or_uci95	Exposure_ID
RCKgN3	TGFrcq	outcome	exposure	MR Egger	8	0.052651	0.037089	0.205535	-0.02004	0.125346	1.054062	0.980156	1.133541	GCST90257015
RCKgN3	TGFrcq	outcome	exposure	Weighted median	8	0.011851	0.024409	0.627309	-0.03599	0.059692	1.011921	0.96465	1.06151	GCST90257015
RCKgN3	TGFrcq	outcome	exposure	Inverse variance weighted	8	-0.01475	0.020875	0.479907	-0.05566	0.026167	0.985361	0.945859	1.026513	GCST90257015
RCKgN3	TGFrcq	outcome	exposure	Simple mode	8	0.015405	0.047145	0.753406	-0.077	0.107809	1.015524	0.925891	1.113835	GCST90257015
RCKgN3	TGFrcq	outcome	exposure	Weighted mode	8	0.016995	0.027633	0.558003	-0.03717	0.071157	1.01714	0.963515	1.073749	GCST90257015
732aJ9	sgTfPU	outcome	exposure	MR Egger	9	-0.02012	0.106732	0.855838	-0.22931	0.189077	0.980083	0.79508	1.208133	GCST90257016
732aJ9	sgTfPU	outcome	exposure	Weighted median	9	-0.0203	0.028974	0.483565	-0.07709	0.03649	0.979906	0.925809	1.037164	GCST90257016
732aJ9	sgTfPU	outcome	exposure	Inverse variance weighted	9	-0.01804	0.023066	0.434246	-0.06324	0.027173	0.982126	0.938714	1.027545	GCST90257016
732aJ9	sgTfPU	outcome	exposure	Simple mode	9	-0.01901	0.043301	0.672185	-0.10388	0.065855	0.981165	0.901329	1.068072	GCST90257016
732aJ9	sgTfPU	outcome	exposure	Weighted mode	9	-0.01783	0.043424	0.692158	-0.10294	0.067283	0.982329	0.90218	1.069598	GCST90257016
poF5dM	SPMYhD	outcome	exposure	MR Egger	14	0.065573	0.057094	0.273139	-0.04633	0.177478	1.067771	0.954725	1.194201	GCST90257017
poF5dM	SPMYhD	outcome	exposure	Weighted median	14	0.020714	0.021323	0.331338	-0.02108	0.062508	1.02093	0.979141	1.064503	GCST90257017
poF5dM	SPMYhD	outcome	exposure	Inverse variance weighted	14	0.020114	0.015434	0.192493	-0.01014	0.050364	1.020317	0.989915	1.051654	GCST90257017
poF5dM	SPMYhD	outcome	exposure	Simple mode	14	0.017974	0.033512	0.60078	-0.04771	0.083657	1.018136	0.953411	1.087256	GCST90257017
poF5dM	SPMYhD	outcome	exposure	Weighted mode	14	0.025411	0.029644	0.406845	-0.03269	0.083513	1.025737	0.967838	1.087099	GCST90257017

id.exposure	id.outcome	outcome	exposure	method	nsnp	b	se	pval	lo_ci	up_ci	or	or_lci95	or_uci95	Exposure_ID
tPgePN	o1ZCMW	outcome	exposure	MR Egger	9	0.004657	0.034576	0.89664	-0.06311	0.072427	1.004668	0.938838	1.075114	GCST90257018
tPgePN	o1ZCMW	outcome	exposure	Weighted median	9	-0.0138	0.012587	0.272891	-0.03847	0.01087	0.986294	0.962258	1.010929	GCST90257018
tPgePN	o1ZCMW	outcome	exposure	Inverse variance weighted	9	-0.01137	0.009625	0.237452	-0.03023	0.007494	0.988694	0.970218	1.007522	GCST90257018
tPgePN	o1ZCMW	outcome	exposure	Simple mode	9	-0.02301	0.018836	0.256717	-0.05993	0.013913	0.977257	0.941835	1.01401	GCST90257018
tPgePN	o1ZCMW	outcome	exposure	Weighted mode	9	-0.01794	0.019241	0.378299	-0.05566	0.019767	0.982216	0.945865	1.019964	GCST90257018
vIjfF4	d1GF2u	outcome	exposure	MR Egger	8	0.096622	0.066098	0.194109	-0.03293	0.226175	1.101444	0.967606	1.253795	GCST90257019
vIjfF4	d1GF2u	outcome	exposure	Weighted median	8	-0.00211	0.026609	0.936823	-0.05426	0.050044	0.997893	0.947184	1.051317	GCST90257019
vIjfF4	d1GF2u	outcome	exposure	Inverse variance weighted	8	-0.00925	0.023446	0.69325	-0.0552	0.036706	0.990794	0.946293	1.037388	GCST90257019
vIjfF4	d1GF2u	outcome	exposure	Simple mode	8	-0.06391	0.052058	0.259241	-0.16595	0.038123	0.938088	0.847092	1.038859	GCST90257019
vIjfF4	d1GF2u	outcome	exposure	Weighted mode	8	0.025655	0.043087	0.5703	-0.05879	0.110105	1.025987	0.9429	1.116395	GCST90257019
B2khrQ	6ekDKh	outcome	exposure	MR Egger	11	0.061886	0.071259	0.407696	-0.07778	0.201553	1.063841	0.925167	1.223301	GCST90257020
B2khrQ	6ekDKh	outcome	exposure	Weighted median	11	0.019964	0.022869	0.382696	-0.02486	0.064787	1.020164	0.975446	1.066932	GCST90257020
B2khrQ	6ekDKh	outcome	exposure	Inverse variance weighted	11	0.012175	0.017804	0.494095	-0.02272	0.04707	1.012249	0.977535	1.048196	GCST90257020
B2khrQ	6ekDKh	outcome	exposure	Simple mode	11	0.009056	0.042876	0.836956	-0.07498	0.093093	1.009097	0.927762	1.097563	GCST90257020
B2khrQ	6ekDKh	outcome	exposure	Weighted mode	11	0.011418	0.038317	0.771806	-0.06368	0.086519	1.011484	0.938303	1.090372	GCST90257020
TanDK9	l4SHxG	outco	exposu	MR Egger	7	-	0.0821	0.9248	-	0.1528	0.9918	0.8443	1.1651	GCST90257

id.exposure	id.outcome	outcome	exposure	method	nsnp	b	se	pval	lo_ci	up_ci	or	or_lci95	or_uci95	Exposure_ID
		me	re			0.00815	63	4	0.16919	9	83	49	97	021
TanDK9	l4SHxG	outcome	exposure	Weighted median	7	0.017593	0.025293	0.486713	-0.03198	0.067168	1.017748	0.968524	1.069475	GCST90257021
TanDK9	l4SHxG	outcome	exposure	Inverse variance weighted	7	0.017431	0.025312	0.491053	-0.03218	0.067042	1.017583	0.968332	1.06934	GCST90257021
TanDK9	l4SHxG	outcome	exposure	Simple mode	7	0.016346	0.042454	0.713494	-0.06686	0.099555	1.01648	0.935323	1.10468	GCST90257021
TanDK9	l4SHxG	outcome	exposure	Weighted mode	7	0.016346	0.02766	0.576116	-0.03787	0.07056	1.01648	0.96284	1.073109	GCST90257021
CpVEyS	XweWyq	outcome	exposure	MR Egger	8	0.097743	0.054634	0.123814	-0.00934	0.204826	1.10268	0.990704	1.227311	GCST90257022
CpVEyS	XweWyq	outcome	exposure	Weighted median	8	0.002057	0.0113	0.855564	-0.02009	0.024205	1.002059	0.980109	1.024501	GCST90257022
CpVEyS	XweWyq	outcome	exposure	Inverse variance weighted	8	0.005301	0.01253	0.672252	-0.01926	0.02986	1.005315	0.980926	1.03031	GCST90257022
CpVEyS	XweWyq	outcome	exposure	Simple mode	8	0.008118	0.016821	0.644111	-0.02485	0.041086	1.008151	0.975455	1.041942	GCST90257022
CpVEyS	XweWyq	outcome	exposure	Weighted mode	8	0.006438	0.015811	0.69604	-0.02455	0.037427	1.006459	0.975748	1.038136	GCST90257022
oB9Uuq	q2o933	outcome	exposure	MR Egger	11	-0.05204	0.063485	0.433551	-0.17647	0.072392	0.949291	0.838223	1.075076	GCST90257023
oB9Uuq	q2o933	outcome	exposure	Weighted median	11	-0.02308	0.022666	0.308459	-0.06751	0.021341	0.97718	0.934718	1.02157	GCST90257023
oB9Uuq	q2o933	outcome	exposure	Inverse variance weighted	11	-0.0223	0.016406	0.174019	-0.05446	0.009853	0.977944	0.946998	1.009902	GCST90257023
oB9Uuq	q2o933	outcome	exposure	Simple mode	11	-0.0076	0.034596	0.830447	-0.07541	0.060203	0.992425	0.927362	1.062052	GCST90257023
oB9Uuq	q2o933	outcome	exposure	Weighted mode	11	-0.00901	0.033484	0.79338	-0.07464	0.056621	0.991032	0.92808	1.058255	GCST90257023
qsD6bK	RVl88S	outcome	exposure	MR Egger	13	-0.03955	0.042784	0.375154	-0.1234	0.04431	0.961226	0.883908	1.045307	GCST90257024

id.exposure	id.outcome	outcome	exposure	method	nsnp	b	se	pval	lo_ci	up_ci	or	or_lci95	or_uci95	Exposure_ID
qsD6bK	RVl88S	outcome	exposure	Weighted median	13	-0.00129	0.010031	0.897335	-0.02096	0.018367	0.998707	0.979262	1.018537	GCST90257024
qsD6bK	RVl88S	outcome	exposure	Inverse variance weighted	13	0.003263	0.009503	0.731355	-0.01536	0.021888	1.003268	0.984755	1.022129	GCST90257024
qsD6bK	RVl88S	outcome	exposure	Simple mode	13	########	0.015526	0.995027	-0.03053	0.030333	0.999901	0.969931	1.030798	GCST90257024
qsD6bK	RVl88S	outcome	exposure	Weighted mode	13	-0.00216	0.013563	0.876372	-0.02874	0.024428	0.997847	0.97167	1.024729	GCST90257024
x94SNo	EpsAdB	outcome	exposure	MR Egger	9	-0.00877	0.033598	0.801602	-0.07462	0.057083	0.991269	0.928095	1.058743	GCST90257025
x94SNo	EpsAdB	outcome	exposure	Weighted median	9	-0.01014	0.013803	0.462409	-0.0372	0.016911	0.989907	0.963485	1.017054	GCST90257025
x94SNo	EpsAdB	outcome	exposure	Inverse variance weighted	9	-0.01022	0.010419	0.326731	-0.03064	0.010203	0.989834	0.969824	1.010256	GCST90257025
x94SNo	EpsAdB	outcome	exposure	Simple mode	9	0.00087	0.02028	0.966828	-0.03888	0.040619	1.000871	0.961867	1.041455	GCST90257025
x94SNo	EpsAdB	outcome	exposure	Weighted mode	9	-0.00774	0.01832	0.683921	-0.04364	0.028171	0.992293	0.957294	1.028572	GCST90257025
0LBV3X	QhyBJ0	outcome	exposure	MR Egger	11	-0.02525	0.017597	0.18518	-0.05974	0.009243	0.975069	0.942011	1.009286	GCST90257026
0LBV3X	QhyBJ0	outcome	exposure	Weighted median	11	0.005147	0.008898	0.562971	-0.01229	0.022587	1.00516	0.987782	1.022844	GCST90257026
0LBV3X	QhyBJ0	outcome	exposure	Inverse variance weighted	11	-0.00228	0.006313	0.717999	-0.01465	0.010094	0.997723	0.985453	1.010145	GCST90257026
0LBV3X	QhyBJ0	outcome	exposure	Simple mode	11	0.012984	0.01569	0.427239	-0.01777	0.043736	1.013069	0.982389	1.044707	GCST90257026
0LBV3X	QhyBJ0	outcome	exposure	Weighted mode	11	0.012984	0.015822	0.430977	-0.01803	0.043995	1.013069	0.982135	1.044977	GCST90257026
xbSVTB	Wkmc0f	outcome	exposure	MR Egger	7	0.012514	0.130858	0.927527	-0.24397	0.268995	1.012593	0.783514	1.308649	GCST90257027
xbSVTB	Wkmc0f	outco	exposu	Weighted median	7	-0.015	0.0269	0.5776	-	0.0378	0.9851	0.9344	1.0385	GCST90257

id.exposure	id.outcome	outcome	exposure	method	nsnp	b	se	pval	lo_ci	up_ci	or	or_lci95	or_uci95	Exposure_ID
		me	re				44	37	0.06781	06	09	35	3	027
xbSVTB	Wkmc0f	outcome	exposure	Inverse variance weighted	7	-0.01727	0.031057	0.578069	-0.07814	0.043597	0.982874	0.92483	1.044561	GCST90257027
xbSVTB	Wkmc0f	outcome	exposure	Simple mode	7	-0.06529	0.059947	0.317914	-0.18278	0.052209	0.936798	0.832949	1.053596	GCST90257027
xbSVTB	Wkmc0f	outcome	exposure	Weighted mode	7	0.030509	0.044674	0.52014	-0.05705	0.11807	1.030979	0.944545	1.125323	GCST90257027
dNzqHF	bbHziK	outcome	exposure	MR Egger	4	-0.17067	0.213881	0.508582	-0.58988	0.248537	0.843099	0.554395	1.282148	GCST90257028
dNzqHF	bbHziK	outcome	exposure	Weighted median	4	-0.03497	0.037552	0.351753	-0.10857	0.038634	0.965636	0.897115	1.03939	GCST90257028
dNzqHF	bbHziK	outcome	exposure	Inverse variance weighted	4	-0.01905	0.040923	0.641616	-0.09926	0.061162	0.981133	0.905511	1.063071	GCST90257028
dNzqHF	bbHziK	outcome	exposure	Simple mode	4	-0.04236	0.054075	0.49058	-0.14835	0.063628	0.958525	0.862132	1.065696	GCST90257028
dNzqHF	bbHziK	outcome	exposure	Weighted mode	4	-0.04394	0.053042	0.468186	-0.1479	0.060022	0.95701	0.862514	1.06186	GCST90257028
nrJFcn	shU5KC	outcome	exposure	MR Egger	7	0.093352	0.103792	0.409656	-0.11008	0.296784	1.097848	0.895762	1.345524	GCST90257029
nrJFcn	shU5KC	outcome	exposure	Weighted median	7	-0.01339	0.02684	0.61778	-0.066	0.039213	0.986696	0.936131	1.039992	GCST90257029
nrJFcn	shU5KC	outcome	exposure	Inverse variance weighted	7	-0.00567	0.023327	0.807826	-0.05139	0.040046	0.994342	0.949904	1.040859	GCST90257029
nrJFcn	shU5KC	outcome	exposure	Simple mode	7	-0.04536	0.047688	0.378199	-0.13883	0.048104	0.95565	0.870375	1.04928	GCST90257029
nrJFcn	shU5KC	outcome	exposure	Weighted mode	7	-0.04089	0.048032	0.427289	-0.13503	0.053255	0.959937	0.87369	1.054698	GCST90257029
UfFDqX	LySwPX	outcome	exposure	MR Egger	10	0.100965	0.094768	0.317797	-0.08478	0.286711	1.106238	0.918713	1.332039	GCST90257030
UfFDqX	LySwPX	outcome	exposure	Weighted median	10	0.005812	0.02629	0.825042	-0.04572	0.05734	1.005829	0.955313	1.059015	GCST90257030

id.exposure	id.outcome	outcome	exposure	method	nsnp	b	se	pval	lo_ci	up_ci	or	or_lci95	or_uci95	Exposure_ID
UfFDqX	LySwPX	outcome	exposure	Inverse variance weighted	10	0.0121	0.019143	0.527328	-0.02542	0.04962	1.012174	0.9749	1.050872	GCST90257030
UfFDqX	LySwPX	outcome	exposure	Simple mode	10	-0.00023	0.041648	0.995797	-0.08186	0.081405	0.999774	0.921405	1.08481	GCST90257030
UfFDqX	LySwPX	outcome	exposure	Weighted mode	10	0.001093	0.041136	0.979389	-0.07953	0.081718	1.001093	0.923547	1.08515	GCST90257030
deRug4	iSJF8v	outcome	exposure	MR Egger	6	-0.01736	0.053276	0.760824	-0.12178	0.087057	0.982786	0.885339	1.090959	GCST90257031
deRug4	iSJF8v	outcome	exposure	Weighted median	6	-0.00208	0.015365	0.892348	-0.03219	0.028036	0.997923	0.968318	1.028433	GCST90257031
deRug4	iSJF8v	outcome	exposure	Inverse variance weighted	6	0.00668	0.011793	0.571062	-0.01643	0.029794	1.006703	0.983701	1.030242	GCST90257031
deRug4	iSJF8v	outcome	exposure	Simple mode	6	-0.00356	0.021105	0.872614	-0.04493	0.037804	0.996445	0.956068	1.038527	GCST90257031
deRug4	iSJF8v	outcome	exposure	Weighted mode	6	-0.00424	0.020648	0.845456	-0.04471	0.036232	0.99577	0.956275	1.036897	GCST90257031
070U1w	yqjZ64	outcome	exposure	MR Egger	13	0.080129	0.093391	0.409199	-0.10292	0.263175	1.083427	0.902202	1.301054	GCST90257032
070U1w	yqjZ64	outcome	exposure	Weighted median	13	-0.01627	0.022518	0.469966	-0.06041	0.027865	0.983862	0.941383	1.028257	GCST90257032
070U1w	yqjZ64	outcome	exposure	Inverse variance weighted	13	0.004448	0.019445	0.819083	-0.03367	0.04256	1.004458	0.966895	1.043479	GCST90257032
070U1w	yqjZ64	outcome	exposure	Simple mode	13	-0.03702	0.041041	0.384731	-0.11746	0.043415	0.963652	0.889172	1.044371	GCST90257032
070U1w	yqjZ64	outcome	exposure	Weighted mode	13	-0.03255	0.040568	0.437939	-0.11207	0.046963	0.967973	0.893986	1.048083	GCST90257032
vRcwW3	srhV00	outcome	exposure	MR Egger	7	-0.04201	0.063321	0.536431	-0.16612	0.082101	0.958863	0.846949	1.085566	GCST90257033
vRcwW3	srhV00	outcome	exposure	Weighted median	7	0.020483	0.026899	0.44636	-0.03224	0.073205	1.020695	0.968276	1.075951	GCST90257033
vRcwW3	srhV00	outco	exposu	Inverse variance	7	0.03525	0.0277	0.2046	-	0.0897	1.0358	0.9809	1.0938	GCST90257

id.exposure	id.outcome	outcome	exposure	method	nsnp	b	se	pval	lo_ci	up_ci	or	or_lci95	or_uci95	Exposure_ID
		me	re	weighted		8	96	27	0.01922	38	87	62	87	033
vRcwW3	srhV00	outcome	exposure	Simple mode	7	0.016834	0.038893	0.680265	-0.0594	0.093065	1.016977	0.942333	1.097533	GCST90257033
vRcwW3	srhV00	outcome	exposure	Weighted mode	7	0.01896	0.02833	0.528217	-0.03657	0.074487	1.019141	0.964095	1.077331	GCST90257033
iqNP8V	gG8Cea	outcome	exposure	MR Egger	8	-0.04657	0.038195	0.268529	-0.12143	0.028296	0.954502	0.885655	1.0287	GCST90257034
iqNP8V	gG8Cea	outcome	exposure	Weighted median	8	-0.01706	0.013582	0.209005	-0.04368	0.009558	0.983082	0.957256	1.009603	GCST90257034
iqNP8V	gG8Cea	outcome	exposure	Inverse variance weighted	8	-0.01846	0.010054	0.066396	-0.03816	0.001249	0.981713	0.962557	1.00125	GCST90257034
iqNP8V	gG8Cea	outcome	exposure	Simple mode	8	-0.01896	0.020697	0.390074	-0.05953	0.021605	0.981218	0.942211	1.02184	GCST90257034
iqNP8V	gG8Cea	outcome	exposure	Weighted mode	8	-0.02195	0.019528	0.298083	-0.06022	0.016326	0.97829	0.941554	1.01646	GCST90257034
qhOwXz	8oS8YC	outcome	exposure	MR Egger	10	0.007061	0.026816	0.798971	-0.0455	0.059621	1.007086	0.955521	1.061434	GCST90257035
qhOwXz	8oS8YC	outcome	exposure	Weighted median	10	0.005812	0.007014	0.407299	-0.00794	0.01956	1.005829	0.992096	1.019752	GCST90257035
qhOwXz	8oS8YC	outcome	exposure	Inverse variance weighted	10	0.008302	0.008054	0.302674	-0.00748	0.024088	1.008336	0.992543	1.02438	GCST90257035
qhOwXz	8oS8YC	outcome	exposure	Simple mode	10	0.020568	0.013981	0.175325	-0.00683	0.04797	1.020781	0.993189	1.04914	GCST90257035
qhOwXz	8oS8YC	outcome	exposure	Weighted mode	10	0.011726	0.01226	0.363812	-0.0123	0.035755	1.011795	0.987773	1.036402	GCST90257035
K3qMLD	q1Q3ER	outcome	exposure	MR Egger	9	0.107998	0.132759	0.442727	-0.15221	0.368206	1.114045	0.858807	1.44514	GCST90257036
K3qMLD	q1Q3ER	outcome	exposure	Weighted median	9	-0.04437	0.026285	0.091396	-0.09589	0.007148	0.956598	0.908563	1.007173	GCST90257036
K3qMLD	q1Q3ER	outcome	exposure	Inverse variance weighted	9	-0.00874	0.028307	0.757614	-0.06422	0.046746	0.991302	0.937801	1.047856	GCST90257036

id.exposure	id.outcome	outcome	exposure	method	nsnp	b	se	pval	lo_ci	up_ci	or	or_lci95	or_uci95	Exposure_ID
K3qMLD	q1Q3ER	outcome	exposure	Simple mode	9	-0.05914	0.036878	0.147436	-0.13142	0.013138	0.942571	0.876845	1.013224	GCST90257036
K3qMLD	q1Q3ER	outcome	exposure	Weighted mode	9	-0.05583	0.033051	0.129635	-0.12061	0.008947	0.945698	0.886378	1.008987	GCST90257036
eeEoF8	GJG3mb	outcome	exposure	MR Egger	10	0.067489	0.074279	0.390121	-0.0781	0.213076	1.069818	0.924873	1.237479	GCST90257037
eeEoF8	GJG3mb	outcome	exposure	Weighted median	10	-0.00869	0.025594	0.734182	-0.05885	0.041473	0.991347	0.942844	1.042345	GCST90257037
eeEoF8	GJG3mb	outcome	exposure	Inverse variance weighted	10	-0.00791	0.021594	0.714006	-0.05024	0.034411	0.992117	0.951002	1.03501	GCST90257037
eeEoF8	GJG3mb	outcome	exposure	Simple mode	10	-0.02463	0.044862	0.596357	-0.11256	0.0633	0.975671	0.893545	1.065346	GCST90257037
eeEoF8	GJG3mb	outcome	exposure	Weighted mode	10	-0.01853	0.04398	0.683346	-0.10473	0.067668	0.981637	0.900563	1.07001	GCST90257037
fs9Rle	LX2glv	outcome	exposure	MR Egger	13	-0.05947	0.070534	0.417134	-0.19771	0.07878	0.942267	0.820606	1.081966	GCST90257038
fs9Rle	LX2glv	outcome	exposure	Weighted median	13	-0.00796	0.01938	0.68138	-0.04594	0.030028	0.992074	0.955097	1.030483	GCST90257038
fs9Rle	LX2glv	outcome	exposure	Inverse variance weighted	13	-0.01659	0.01515	0.27355	-0.04628	0.013106	0.983549	0.954772	1.013192	GCST90257038
fs9Rle	LX2glv	outcome	exposure	Simple mode	13	-0.00255	0.034122	0.941693	-0.06943	0.06433	0.997455	0.932928	1.066444	GCST90257038
fs9Rle	LX2glv	outcome	exposure	Weighted mode	13	-0.00055	0.031528	0.986369	-0.06235	0.061245	0.99945	0.939559	1.06316	GCST90257038
7dWHtK	oyzoUc	outcome	exposure	MR Egger	10	0.023417	0.028749	0.438908	-0.03293	0.079764	1.023693	0.967606	1.083032	GCST90257039
7dWHtK	oyzoUc	outcome	exposure	Weighted median	10	-0.00705	0.008208	0.390682	-0.02313	0.009042	0.992979	0.977133	1.009083	GCST90257039
7dWHtK	oyzoUc	outcome	exposure	Inverse variance weighted	10	-0.00625	0.005633	0.267275	-0.01729	0.004791	0.993771	0.98286	1.004803	GCST90257039
7dWHtK	oyzoUc	outco	exposu	Simple mode	10	-	0.0127	0.4916	-	0.0159	0.9908	0.9663	1.0160	GCST90257

id.exposure	id.outcome	outcome	exposure	method	nsnp	b	se	pval	lo_ci	up_ci	or	or_lci95	or_uci95	Exposure_ID
		me	re			0.00917	91	73	0.03424	02	73	39	29	039
7dWHtK	oyzoUc	outcome	exposure	Weighted mode	10	-0.00835	0.012601	0.524006	-0.03305	0.016344	0.991682	0.967489	1.016479	GCST90257039
Y0nGkJ	IZ5OXY	outcome	exposure	MR Egger	6	-0.17608	0.248037	0.516963	-0.66223	0.310072	0.838551	0.515699	1.363524	GCST90257040
Y0nGkJ	IZ5OXY	outcome	exposure	Weighted median	6	0.052163	0.026133	0.045928	0.000942	0.103383	1.053547	1.000943	1.108916	GCST90257040
Y0nGkJ	IZ5OXY	outcome	exposure	Inverse variance weighted	6	0.063932	0.027453	0.019873	0.010123	0.11774	1.066019	1.010175	1.124952	GCST90257040
Y0nGkJ	IZ5OXY	outcome	exposure	Simple mode	6	0.069674	0.042801	0.164482	-0.01422	0.153564	1.072159	0.985885	1.165982	GCST90257040
Y0nGkJ	IZ5OXY	outcome	exposure	Weighted mode	6	0.052537	0.033264	0.175075	-0.01266	0.117734	1.053942	0.98742	1.124945	GCST90257040
FstY1C	10Jfz7	outcome	exposure	MR Egger	10	-0.0357	0.046948	0.468782	-0.12772	0.056315	0.964927	0.880099	1.057931	GCST90257041
FstY1C	10Jfz7	outcome	exposure	Weighted median	10	-0.01237	0.01731	0.474907	-0.0463	0.02156	0.987708	0.954759	1.021794	GCST90257041
FstY1C	10Jfz7	outcome	exposure	Inverse variance weighted	10	-0.00988	0.014327	0.490585	-0.03796	0.018204	0.990172	0.962753	1.018371	GCST90257041
FstY1C	10Jfz7	outcome	exposure	Simple mode	10	0.014113	0.028783	0.63564	-0.0423	0.070528	1.014213	0.95858	1.073074	GCST90257041
FstY1C	10Jfz7	outcome	exposure	Weighted mode	10	-0.01558	0.019841	0.452497	-0.05447	0.023308	0.984541	0.94699	1.023582	GCST90257041
F1p652	3kHJyA	outcome	exposure	MR Egger	7	0.064096	0.033499	0.113895	-0.00156	0.129755	1.066195	0.998439	1.138549	GCST90257042
F1p652	3kHJyA	outcome	exposure	Weighted median	7	0.02194	0.014052	0.118447	-0.0056	0.049482	1.022182	0.994413	1.050727	GCST90257042
F1p652	3kHJyA	outcome	exposure	Inverse variance weighted	7	0.011126	0.013917	0.42403	-0.01615	0.038403	1.011188	0.983978	1.03915	GCST90257042
F1p652	3kHJyA	outcome	exposure	Simple mode	7	0.017539	0.025168	0.511964	-0.03179	0.066867	1.017693	0.96871	1.069153	GCST90257042

id.exposure	id.outcome	outcome	exposure	method	nsnp	b	se	pval	lo_ci	up_ci	or	or_lci95	or_uci95	Exposure_ID
F1p652	3kHJyA	outcome	exposure	Weighted mode	7	0.026411	0.01909	0.215789	-0.01101	0.063828	1.026763	0.989055	1.065909	GCST90257042
1y3Sqg	bOoox2	outcome	exposure	MR Egger	9	-0.03054	0.0822	0.721231	-0.19165	0.130572	0.969921	0.825594	1.13948	GCST90257043
1y3Sqg	bOoox2	outcome	exposure	Weighted median	9	-0.00239	0.02196	0.91317	-0.04544	0.040647	0.997608	0.955581	1.041484	GCST90257043
1y3Sqg	bOoox2	outcome	exposure	Inverse variance weighted	9	0.002341	0.018471	0.899154	-0.03386	0.038544	1.002344	0.966705	1.039296	GCST90257043
1y3Sqg	bOoox2	outcome	exposure	Simple mode	9	0.000809	0.03844	0.983726	-0.07453	0.076152	1.000809	0.928176	1.079126	GCST90257043
1y3Sqg	bOoox2	outcome	exposure	Weighted mode	9	-0.00172	0.025691	0.948188	-0.05208	0.048631	0.998279	0.949256	1.049833	GCST90257043
SeCFZc	QMfSVB	outcome	exposure	MR Egger	13	0.073686	0.085734	0.408416	-0.09435	0.241724	1.076469	0.909963	1.273443	GCST90257044
SeCFZc	QMfSVB	outcome	exposure	Weighted median	13	0.008258	0.024014	0.730933	-0.03881	0.055325	1.008292	0.961935	1.056884	GCST90257044
SeCFZc	QMfSVB	outcome	exposure	Inverse variance weighted	13	0.024729	0.022545	0.272702	-0.01946	0.068917	1.025037	0.980729	1.071347	GCST90257044
SeCFZc	QMfSVB	outcome	exposure	Simple mode	13	-0.00513	0.038397	0.895887	-0.08039	0.070127	0.994881	0.922755	1.072644	GCST90257044
SeCFZc	QMfSVB	outcome	exposure	Weighted mode	13	0.009527	0.029432	0.751741	-0.04816	0.067214	1.009573	0.952981	1.069524	GCST90257044
5cwTtU	vX2IV3	outcome	exposure	MR Egger	8	-0.01184	0.099202	0.908889	-0.20628	0.182596	0.988229	0.813608	1.200329	GCST90257045
5cwTtU	vX2IV3	outcome	exposure	Weighted median	8	0.01034	0.027606	0.707991	-0.04377	0.064447	1.010394	0.957176	1.066569	GCST90257045
5cwTtU	vX2IV3	outcome	exposure	Inverse variance weighted	8	-0.00166	0.020837	0.936478	-0.0425	0.039179	0.998341	0.95839	1.039957	GCST90257045
5cwTtU	vX2IV3	outcome	exposure	Simple mode	8	0.024104	0.050618	0.648448	-0.07511	0.123315	1.024396	0.927643	1.131241	GCST90257045
5cwTtU	vX2IV3	outco	exposu	Weighted mode	8	0.02410	0.0516	0.6546	-	0.1252	1.0243	0.9258	1.1334	GCST90257

id.exposure	id.outcome	outcome	exposure	method	nsnp	b	se	pval	lo_ci	up_ci	or	or_lci95	or_uci95	Exposure_ID
		me	re			4	02	18	0.07704	44	96	56	25	045
juymMs	Vgfy1t	outcome	exposure	MR Egger	10	-0.0717	0.070364	0.338061	-0.20961	0.066218	0.930814	0.810902	1.068459	GCST90257046
juymMs	Vgfy1t	outcome	exposure	Weighted median	10	0.000202	0.029119	0.994463	-0.05687	0.057276	1.000202	0.944716	1.058948	GCST90257046
juymMs	Vgfy1t	outcome	exposure	Inverse variance weighted	10	-0.00843	0.021723	0.697816	-0.05101	0.034142	0.991601	0.950269	1.034731	GCST90257046
juymMs	Vgfy1t	outcome	exposure	Simple mode	10	0.019316	0.041579	0.65328	-0.06218	0.100812	1.019504	0.939714	1.106069	GCST90257046
juymMs	Vgfy1t	outcome	exposure	Weighted mode	10	0.008232	0.039914	0.841199	-0.07	0.086463	1.008266	0.932394	1.090311	GCST90257046
SqUVHt	LoMOp0	outcome	exposure	MR Egger	7	0.117918	0.086868	0.232696	-0.05234	0.288179	1.125152	0.949003	1.333996	GCST90257047
SqUVHt	LoMOp0	outcome	exposure	Weighted median	7	-0.02208	0.031033	0.476776	-0.08291	0.038745	0.978162	0.920438	1.039506	GCST90257047
SqUVHt	LoMOp0	outcome	exposure	Inverse variance weighted	7	-0.02102	0.023326	0.367581	-0.06674	0.024702	0.979202	0.935442	1.025009	GCST90257047
SqUVHt	LoMOp0	outcome	exposure	Simple mode	7	-0.03692	0.046472	0.457235	-0.128	0.054169	0.963757	0.879851	1.055663	GCST90257047
SqUVHt	LoMOp0	outcome	exposure	Weighted mode	7	-0.05225	0.043058	0.270505	-0.13665	0.032139	0.949088	0.872278	1.032661	GCST90257047
5T9g6W	Hxw76j	outcome	exposure	MR Egger	4	-0.12866	0.250188	0.658266	-0.61903	0.36171	0.879275	0.538469	1.435783	GCST90257048
5T9g6W	Hxw76j	outcome	exposure	Weighted median	4	-0.04206	0.036593	0.250427	-0.11378	0.029665	0.958816	0.892456	1.03011	GCST90257048
5T9g6W	Hxw76j	outcome	exposure	Inverse variance weighted	4	-0.03153	0.031273	0.313354	-0.09282	0.029765	0.968963	0.911354	1.030212	GCST90257048
5T9g6W	Hxw76j	outcome	exposure	Simple mode	4	-0.04592	0.047275	0.403048	-0.13858	0.046742	0.95512	0.870596	1.047852	GCST90257048
5T9g6W	Hxw76j	outcome	exposure	Weighted mode	4	-0.0469	0.046733	0.389544	-0.13849	0.044699	0.954185	0.870668	1.045713	GCST90257048

id.exposure	id.outcome	outcome	exposure	method	nsnp	b	se	pval	lo_ci	up_ci	or	or_lci95	or_uci95	Exposure_ID
yMlKz9	EGtStV	outcome	exposure	MR Egger	10	0.037528	0.105169	0.730446	-0.1686	0.243658	1.038241	0.844844	1.275908	GCST90257049
yMlKz9	EGtStV	outcome	exposure	Weighted median	10	-0.02595	0.027081	0.33799	-0.07903	0.027131	0.974387	0.924016	1.027502	GCST90257049
yMlKz9	EGtStV	outcome	exposure	Inverse variance weighted	10	-0.00955	0.02174	0.660557	-0.05216	0.033063	0.990499	0.94918	1.033616	GCST90257049
yMlKz9	EGtStV	outcome	exposure	Simple mode	10	-0.03357	0.045822	0.482427	-0.12338	0.056241	0.966986	0.883926	1.057852	GCST90257049
yMlKz9	EGtStV	outcome	exposure	Weighted mode	10	-0.03858	0.054386	0.496049	-0.14518	0.068015	0.962154	0.86487	1.070381	GCST90257049
fD9Ybz	Wr9ZQG	outcome	exposure	MR Egger	12	-0.07338	0.045823	0.140357	-0.1632	0.016429	0.929245	0.849425	1.016565	GCST90257050
fD9Ybz	Wr9ZQG	outcome	exposure	Weighted median	12	-0.01662	0.020628	0.42029	-0.05706	0.023806	0.983513	0.944542	1.024092	GCST90257050
fD9Ybz	Wr9ZQG	outcome	exposure	Inverse variance weighted	12	-0.00245	0.016714	0.883246	-0.03521	0.030304	0.997549	0.9654	1.030768	GCST90257050
fD9Ybz	Wr9ZQG	outcome	exposure	Simple mode	12	-0.04397	0.039046	0.284061	-0.1205	0.032557	0.95698	0.886475	1.033093	GCST90257050
fD9Ybz	Wr9ZQG	outcome	exposure	Weighted mode	12	-0.02401	0.025287	0.36278	-0.07357	0.025553	0.976277	0.92907	1.025883	GCST90257050
y88njx	tBK8Wy	outcome	exposure	MR Egger	11	-0.01025	0.065229	0.878572	-0.1381	0.117597	0.9898	0.871009	1.124791	GCST90257051
y88njx	tBK8Wy	outcome	exposure	Weighted median	11	-0.00963	0.022808	0.67293	-0.05433	0.035075	0.990418	0.947118	1.035698	GCST90257051
y88njx	tBK8Wy	outcome	exposure	Inverse variance weighted	11	-0.02214	0.016941	0.191262	-0.05534	0.011065	0.978104	0.94616	1.011126	GCST90257051
y88njx	tBK8Wy	outcome	exposure	Simple mode	11	-0.00278	0.03537	0.938842	-0.07211	0.066543	0.997221	0.93043	1.068807	GCST90257051
y88njx	tBK8Wy	outcome	exposure	Weighted mode	11	2.61E-05	0.029498	0.99931	-0.05779	0.057842	1.000026	0.943848	1.059548	GCST90257051
jFBpmu	G80M5t	outco	exposu	MR Egger	11	0.03250	0.1743	0.8562	-0.3092	0.3742	1.0330	0.7340	1.4538	GCST90257

id.exposure	id.outcome	outcome	exposure	method	nsnp	b	se	pval	lo_ci	up_ci	or	or_lci95	or_uci95	Exposure_ID
		me	re			4	36	33		03	38	37	32	052
jFBpmu	G80M5t	outcome	exposure	Weighted median	11	0.026615	0.024923	0.285568	-0.02223	0.075464	1.026973	0.978011	1.078385	GCST90257052
jFBpmu	G80M5t	outcome	exposure	Inverse variance weighted	11	0.025883	0.022314	0.246074	-0.01785	0.069619	1.026221	0.982306	1.0721	GCST90257052
jFBpmu	G80M5t	outcome	exposure	Simple mode	11	0.038378	0.042951	0.392562	-0.0458	0.122561	1.039124	0.955228	1.130388	GCST90257052
jFBpmu	G80M5t	outcome	exposure	Weighted mode	11	0.034662	0.040287	0.409735	-0.0443	0.113625	1.03527	0.956666	1.120332	GCST90257052
TlCzSf	OcX0NA	outcome	exposure	MR Egger	17	0.066339	0.099496	0.515058	-0.12867	0.261351	1.068589	0.879261	1.298683	GCST90257053
TlCzSf	OcX0NA	outcome	exposure	Weighted median	17	0.016121	0.0219	0.461668	-0.0268	0.059045	1.016251	0.973552	1.060823	GCST90257053
TlCzSf	OcX0NA	outcome	exposure	Inverse variance weighted	17	0.011378	0.01911	0.551566	-0.02608	0.048834	1.011443	0.97426	1.050046	GCST90257053
TlCzSf	OcX0NA	outcome	exposure	Simple mode	17	0.003931	0.035566	0.913376	-0.06578	0.07364	1.003938	0.936338	1.076419	GCST90257053
TlCzSf	OcX0NA	outcome	exposure	Weighted mode	17	0.005006	0.03045	0.871488	-0.05468	0.064688	1.005018	0.946791	1.066826	GCST90257053
uC0zVw	79ZstP	outcome	exposure	MR Egger	10	0.035292	0.069899	0.627242	-0.10171	0.172293	1.035922	0.903292	1.188026	GCST90257054
uC0zVw	79ZstP	outcome	exposure	Weighted median	10	0.013819	0.025397	0.586348	-0.03596	0.063598	1.013915	0.96468	1.065664	GCST90257054
uC0zVw	79ZstP	outcome	exposure	Inverse variance weighted	10	0.012248	0.019549	0.530964	-0.02607	0.050564	1.012323	0.974269	1.051864	GCST90257054
uC0zVw	79ZstP	outcome	exposure	Simple mode	10	0.010717	0.039328	0.791379	-0.06637	0.0878	1.010775	0.935789	1.091769	GCST90257054
uC0zVw	79ZstP	outcome	exposure	Weighted mode	10	0.011298	0.036249	0.762386	-0.05975	0.082346	1.011362	0.942	1.085831	GCST90257054
5wCNxN	zgK60V	outcome	exposure	MR Egger	15	0.000389	0.026973	0.988706	-0.05248	0.053256	1.000389	0.948876	1.054699	GCST90257055

id.exposure	id.outcome	outcome	exposure	method	nsnp	b	se	pval	lo_ci	up_ci	or	or_lci95	or_uci95	Exposure_ID
5wCNxN	zgK60V	outcome	exposure	Weighted median	15	0.000412	0.008748	0.962468	-0.01674	0.017558	1.000412	0.983404	1.017714	GCST90257055
5wCNxN	zgK60V	outcome	exposure	Inverse variance weighted	15	0.000816	0.006567	0.901135	-0.01205	0.013686	1.000816	0.988018	1.01378	GCST90257055
5wCNxN	zgK60V	outcome	exposure	Simple mode	15	0.003076	0.014075	0.830143	-0.02451	0.030663	1.003081	0.975788	1.031138	GCST90257055
5wCNxN	zgK60V	outcome	exposure	Weighted mode	15	0.001046	0.013371	0.938775	-0.02516	0.027253	1.001046	0.975152	1.027627	GCST90257055
3Qv8UW	ePhOaP	outcome	exposure	MR Egger	8	0.014794	0.04341	0.744871	-0.07029	0.099877	1.014904	0.932124	1.105035	GCST90257056
3Qv8UW	ePhOaP	outcome	exposure	Weighted median	8	-0.01371	0.012014	0.253763	-0.03726	0.009837	0.986382	0.963426	1.009885	GCST90257056
3Qv8UW	ePhOaP	outcome	exposure	Inverse variance weighted	8	-0.00278	0.009565	0.771618	-0.02152	0.015971	0.997228	0.978706	1.016099	GCST90257056
3Qv8UW	ePhOaP	outcome	exposure	Simple mode	8	-0.02089	0.020798	0.348721	-0.06165	0.019878	0.979331	0.940213	1.020077	GCST90257056
3Qv8UW	ePhOaP	outcome	exposure	Weighted mode	8	-0.02025	0.020567	0.357669	-0.06056	0.020063	0.979956	0.941239	1.020265	GCST90257056
9pWrcc	oaHvoD	outcome	exposure	MR Egger	6	-0.0724	0.049231	0.215326	-0.1689	0.024088	0.930155	0.844597	1.02438	GCST90257057
9pWrcc	oaHvoD	outcome	exposure	Weighted median	6	0.03477	0.013763	0.011527	0.007794	0.061746	1.035381	1.007824	1.063692	GCST90257057
9pWrcc	oaHvoD	outcome	exposure	Inverse variance weighted	6	0.022215	0.014147	0.116352	-0.00551	0.049943	1.022463	0.994502	1.051211	GCST90257057
9pWrcc	oaHvoD	outcome	exposure	Simple mode	6	0.039065	0.020346	0.112932	-0.00081	0.078944	1.039838	0.999186	1.082144	GCST90257057
9pWrcc	oaHvoD	outcome	exposure	Weighted mode	6	0.039065	0.01997	0.107812	########	0.078205	1.039838	0.999924	1.081345	GCST90257057
v3Su9O	mBtA22	outcome	exposure	MR Egger	5	0.094356	0.064096	0.237385	-0.03127	0.219984	1.098951	0.969213	1.246057	GCST90257058
v3Su9O	mBtA22	outco	exposu	Weighted median	5	-	0.0157	0.4093	-	0.0179	0.9870	0.9569	1.0180	GCST90257

id.exposure	id.outcome	outcome	exposure	method	nsnp	b	se	pval	lo_ci	up_ci	or	or_lci95	or_uci95	Exposure_ID
		me	re			0.01302	83	63	0.04396	13	63	97	75	058
v3Su9O	mBtA22	outcome	exposure	Inverse variance weighted	5	-0.00534	0.012253	0.663197	-0.02935	0.018679	0.994678	0.971075	1.018855	GCST90257058
v3Su9O	mBtA22	outcome	exposure	Simple mode	5	-0.02191	0.023965	0.412231	-0.06889	0.025057	0.978324	0.933434	1.025374	GCST90257058
v3Su9O	mBtA22	outcome	exposure	Weighted mode	5	-0.02403	0.025728	0.403136	-0.07446	0.026395	0.976254	0.928245	1.026746	GCST90257058
hNYdEo	sf2Ymf	outcome	exposure	MR Egger	6	-0.08775	0.077689	0.321821	-0.24002	0.064518	0.915988	0.78661	1.066645	GCST90257059
hNYdEo	sf2Ymf	outcome	exposure	Weighted median	6	0.018045	0.024018	0.452483	-0.02903	0.06512	1.018208	0.971386	1.067288	GCST90257059
hNYdEo	sf2Ymf	outcome	exposure	Inverse variance weighted	6	0.025932	0.018492	0.160812	-0.01031	0.062177	1.026272	0.989741	1.064151	GCST90257059
hNYdEo	sf2Ymf	outcome	exposure	Simple mode	6	0.064273	0.042435	0.190297	-0.0189	0.147446	1.066383	0.981278	1.158871	GCST90257059
hNYdEo	sf2Ymf	outcome	exposure	Weighted mode	6	0.004078	0.027189	0.886643	-0.04921	0.057369	1.004086	0.951978	1.059047	GCST90257059
toAION	fWdeEs	outcome	exposure	MR Egger	8	-0.18822	0.119891	0.167482	-0.42321	0.046765	0.828432	0.654943	1.047876	GCST90257060
toAION	fWdeEs	outcome	exposure	Weighted median	8	-0.02273	0.026213	0.385807	-0.07411	0.028644	0.977523	0.928569	1.029059	GCST90257060
toAION	fWdeEs	outcome	exposure	Inverse variance weighted	8	-0.00698	0.028751	0.808192	-0.06333	0.049373	0.993045	0.938632	1.050612	GCST90257060
toAION	fWdeEs	outcome	exposure	Simple mode	8	-0.03618	0.045446	0.452107	-0.12526	0.052893	0.964466	0.882272	1.054317	GCST90257060
toAION	fWdeEs	outcome	exposure	Weighted mode	8	-0.03718	0.033436	0.302904	-0.10271	0.028357	0.963505	0.902386	1.028763	GCST90257060
HUJIac	i0Up1q	outcome	exposure	MR Egger	7	-0.22751	0.100396	0.072794	-0.42428	-0.03073	0.796517	0.654238	0.969737	GCST90257061
HUJIac	i0Up1q	outco	exposu	Weighted median	7	-	0.0239	0.9812	-	0.0462	0.9994	0.9536	1.0473	GCST90257

id.exposure	id.outcome	outcome	exposure	method	nsnp	b	se	pval	lo_ci	up_ci	or	or_lci95	or_uci95	Exposure_ID
		me	re			0.00056	04	27	0.04741	9	38	92	78	061
HUJIac	i0Up1q	outcome	exposure	Inverse variance weighted	7	-0.02756	0.024471	0.260147	-0.07552	0.020408	0.972821	0.927263	1.020617	GCST90257061
HUJIac	i0Up1q	outcome	exposure	Simple mode	7	0.000254	0.036959	0.994733	-0.07219	0.072695	1.000254	0.930358	1.075402	GCST90257061
HUJIac	i0Up1q	outcome	exposure	Weighted mode	7	0.000958	0.029436	0.975096	-0.05674	0.058653	1.000958	0.944842	1.060407	GCST90257061
xWVCnc	EdtpjT	outcome	exposure	MR Egger	9	0.009656	0.075939	0.902392	-0.13918	0.158496	1.009703	0.870068	1.171748	GCST90257062
xWVCnc	EdtpjT	outcome	exposure	Weighted median	9	0.040447	0.026238	0.123188	-0.01098	0.091874	1.041276	0.98908	1.096227	GCST90257062
xWVCnc	EdtpjT	outcome	exposure	Inverse variance weighted	9	0.026558	0.023476	0.257932	-0.01945	0.07257	1.026914	0.980734	1.075269	GCST90257062
xWVCnc	EdtpjT	outcome	exposure	Simple mode	9	0.068783	0.049856	0.205032	-0.02893	0.166501	1.071204	0.97148	1.181164	GCST90257062
xWVCnc	EdtpjT	outcome	exposure	Weighted mode	9	0.054768	0.032459	0.130029	-0.00885	0.118389	1.056296	0.991187	1.125682	GCST90257062
hHKBaH	UzSEn2	outcome	exposure	MR Egger	11	-0.02206	0.090873	0.813642	-0.20017	0.156053	0.978182	0.818591	1.168888	GCST90257063
hHKBaH	UzSEn2	outcome	exposure	Weighted median	11	-0.00789	0.02113	0.708822	-0.0493	0.033523	0.99214	0.951891	1.034092	GCST90257063
hHKBaH	UzSEn2	outcome	exposure	Inverse variance weighted	11	0.000863	0.01769	0.961108	-0.03381	0.035534	1.000863	0.966756	1.036173	GCST90257063
hHKBaH	UzSEn2	outcome	exposure	Simple mode	11	-0.0339	0.037054	0.381844	-0.10652	0.038731	0.966672	0.898955	1.03949	GCST90257063
hHKBaH	UzSEn2	outcome	exposure	Weighted mode	11	-0.01671	0.027057	0.550726	-0.06974	0.036324	0.983432	0.932638	1.036992	GCST90257063
q3Oewk	2MXZhX	outcome	exposure	MR Egger	5	-0.14907	0.086996	0.185133	-0.31958	0.021443	0.861509	0.726452	1.021675	GCST90257064
q3Oewk	2MXZhX	outcome	exposure	Weighted median	5	-0.02776	0.027818	0.31826	-0.08229	0.02676	0.972618	0.921008	1.027121	GCST90257064

id.exposure	id.outcome	outcome	exposure	method	nsnp	b	se	pval	lo_ci	up_ci	or	or_lci95	or_uci95	Exposure_ID
q3Oewk	2MXZhX	outcome	exposure	Inverse variance weighted	5	-0.02377	0.022886	0.299037	-0.06862	0.02109	0.976513	0.933677	1.021314	GCST90257064
q3Oewk	2MXZhX	outcome	exposure	Simple mode	5	-0.02548	0.038535	0.544607	-0.10101	0.050047	0.974841	0.903926	1.051321	GCST90257064
q3Oewk	2MXZhX	outcome	exposure	Weighted mode	5	-0.0339	0.033123	0.363981	-0.09882	0.031024	0.96667	0.905906	1.03151	GCST90257064
TKgjz5	EqfI9H	outcome	exposure	MR Egger	10	0.001177	0.037065	0.975448	-0.07147	0.073824	1.001178	0.931024	1.076617	GCST90257065
TKgjz5	EqfI9H	outcome	exposure	Weighted median	10	0.00386	0.011869	0.74499	-0.0194	0.027123	1.003868	0.980785	1.027494	GCST90257065
TKgjz5	EqfI9H	outcome	exposure	Inverse variance weighted	10	0.008013	0.008285	0.33349	-0.00823	0.024252	1.008045	0.991807	1.024548	GCST90257065
TKgjz5	EqfI9H	outcome	exposure	Simple mode	10	-0.00054	0.021203	0.980124	-0.0421	0.041015	0.999457	0.958773	1.041868	GCST90257065
TKgjz5	EqfI9H	outcome	exposure	Weighted mode	10	7.31E-05	0.021538	0.997364	-0.04214	0.042289	1.000073	0.958733	1.043195	GCST90257065
0uTITk	OCeKHN	outcome	exposure	MR Egger	8	0.056068	0.036251	0.172908	-0.01498	0.12712	1.057669	0.985127	1.135553	GCST90257066
0uTITk	OCeKHN	outcome	exposure	Weighted median	8	-0.00151	0.011676	0.897049	-0.0244	0.021374	0.99849	0.9759	1.021604	GCST90257066
0uTITk	OCeKHN	outcome	exposure	Inverse variance weighted	8	########	0.008824	0.994961	-0.01735	0.01724	0.999944	0.982798	1.01739	GCST90257066
0uTITk	OCeKHN	outcome	exposure	Simple mode	8	-0.00424	0.019265	0.832037	-0.042	0.033517	0.995768	0.95887	1.034086	GCST90257066
0uTITk	OCeKHN	outcome	exposure	Weighted mode	8	0.004732	0.015864	0.774139	-0.02636	0.035826	1.004743	0.973983	1.036475	GCST90257066
1E4DDe	skuqQs	outcome	exposure	MR Egger	11	-0.04657	0.051672	0.390888	-0.14785	0.054702	0.954494	0.862561	1.056226	GCST90257067
1E4DDe	skuqQs	outcome	exposure	Weighted median	11	-0.007	0.019387	0.718096	-0.045	0.030999	0.993026	0.956001	1.031485	GCST90257067
1E4DDe	skuqQs	outco	exposu	Inverse variance	11	-	0.0165	0.2904	-	0.0149	0.9826	0.9511	1.0150	GCST90257

id.exposure	id.outcome	outcome	exposure	method	nsnp	b	se	pval	lo_ci	up_ci	or	or_lci95	or_uci95	Exposure_ID
		me	re	weighted		0.01753	87	36	0.05004	75	18	87	88	067
1E4DDe	skuqQs	outcome	exposure	Simple mode	11	-0.02717	0.0332	0.432234	-0.09224	0.037903	0.973197	0.911886	1.03863	GCST90257067
1E4DDe	skuqQs	outcome	exposure	Weighted mode	11	-0.01731	0.02475	0.500256	-0.06582	0.031201	0.982839	0.936299	1.031692	GCST90257067
BfQFz5	iNW4eS	outcome	exposure	MR Egger	10	-0.00902	0.068689	0.898805	-0.14365	0.125614	0.991024	0.866194	1.133844	GCST90257068
BfQFz5	iNW4eS	outcome	exposure	Weighted median	10	0.028389	0.021245	0.181461	-0.01325	0.07003	1.028796	0.986836	1.07254	GCST90257068
BfQFz5	iNW4eS	outcome	exposure	Inverse variance weighted	10	0.005048	0.022025	0.818724	-0.03812	0.048218	1.005061	0.962596	1.049399	GCST90257068
BfQFz5	iNW4eS	outcome	exposure	Simple mode	10	0.030729	0.036848	0.425897	-0.04149	0.102951	1.031206	0.959356	1.108438	GCST90257068
BfQFz5	iNW4eS	outcome	exposure	Weighted mode	10	0.027034	0.034169	0.449184	-0.03994	0.094005	1.027403	0.96085	1.098566	GCST90257068
o2xaGN	oftuCu	outcome	exposure	MR Egger	11	-0.036	0.086259	0.68618	-0.20507	0.133065	0.964638	0.81459	1.142324	GCST90257069
o2xaGN	oftuCu	outcome	exposure	Weighted median	11	0.027393	0.020115	0.173252	-0.01203	0.066818	1.027771	0.98804	1.069101	GCST90257069
o2xaGN	oftuCu	outcome	exposure	Inverse variance weighted	11	-0.00302	0.025832	0.907023	-0.05365	0.047614	0.996987	0.947765	1.048766	GCST90257069
o2xaGN	oftuCu	outcome	exposure	Simple mode	11	0.012173	0.037197	0.750219	-0.06073	0.085079	1.012247	0.941075	1.088803	GCST90257069
o2xaGN	oftuCu	outcome	exposure	Weighted mode	11	0.010787	0.028021	0.70833	-0.04413	0.065709	1.010845	0.956825	1.067916	GCST90257069
HBzUN5	v4fnU3	outcome	exposure	MR Egger	6	-0.01434	0.055866	0.810045	-0.12384	0.095155	0.985759	0.883521	1.099829	GCST90257070
HBzUN5	v4fnU3	outcome	exposure	Weighted median	6	-0.01466	0.012703	0.248526	-0.03956	0.01024	0.985448	0.961215	1.010292	GCST90257070
HBzUN5	v4fnU3	outcome	exposure	Inverse variance weighted	6	-0.00724	0.010367	0.484642	-0.02756	0.013074	0.992781	0.972813	1.01316	GCST90257070

id.exposure	id.outcome	outcome	exposure	method	nsnp	b	se	pval	lo_ci	up_ci	or	or_lci95	or_uci95	Exposure_ID
HBzUN5	v4fnU3	outcome	exposure	Simple mode	6	-0.01669	0.017713	0.389354	-0.05141	0.018028	0.983449	0.949893	1.018192	GCST90257070
HBzUN5	v4fnU3	outcome	exposure	Weighted mode	6	-0.01596	0.016577	0.38	-0.04845	0.016536	0.984171	0.952708	1.016673	GCST90257070
RvEm0j	q81PJW	outcome	exposure	MR Egger	10	-0.02961	0.062094	0.646176	-0.15132	0.09209	0.970821	0.859575	1.096464	GCST90257071
RvEm0j	q81PJW	outcome	exposure	Weighted median	10	-0.01357	0.013425	0.311954	-0.03989	0.012738	0.986517	0.960898	1.01282	GCST90257071
RvEm0j	q81PJW	outcome	exposure	Inverse variance weighted	10	-0.0151	0.012409	0.223785	-0.03942	0.009226	0.985017	0.961349	1.009269	GCST90257071
RvEm0j	q81PJW	outcome	exposure	Simple mode	10	-0.01289	0.024228	0.607503	-0.06038	0.034594	0.98719	0.941406	1.035199	GCST90257071
RvEm0j	q81PJW	outcome	exposure	Weighted mode	10	-0.01085	0.023702	0.658003	-0.0573	0.035607	0.98921	0.944307	1.036248	GCST90257071
EVV22C	Kpnj8r	outcome	exposure	MR Egger	16	-0.02939	0.011947	0.027509	-0.05281	-0.00597	0.971037	0.948563	0.994044	GCST90257072
EVV22C	Kpnj8r	outcome	exposure	Weighted median	16	-0.00789	0.0045	0.079621	-0.01671	0.000932	0.992143	0.983431	1.000932	GCST90257072
EVV22C	Kpnj8r	outcome	exposure	Inverse variance weighted	16	-0.00474	0.003848	0.217734	-0.01228	0.002799	0.995269	0.987791	1.002803	GCST90257072
EVV22C	Kpnj8r	outcome	exposure	Simple mode	16	-0.0064	0.007054	0.378656	-0.02022	0.007426	0.993621	0.979979	1.007454	GCST90257072
EVV22C	Kpnj8r	outcome	exposure	Weighted mode	16	-0.00719	0.006356	0.275717	-0.01965	0.005268	0.992836	0.980544	1.005281	GCST90257072
viyN9N	dMRh3L	outcome	exposure	MR Egger	12	-0.00549	0.018169	0.768582	-0.0411	0.030117	0.994522	0.959729	1.030576	GCST90257073
viyN9N	dMRh3L	outcome	exposure	Weighted median	12	-0.0049	0.005848	0.40212	-0.01636	0.006562	0.995113	0.983772	1.006584	GCST90257073
viyN9N	dMRh3L	outcome	exposure	Inverse variance weighted	12	-0.00406	0.004507	0.368129	-0.01289	0.004777	0.995952	0.987193	1.004789	GCST90257073

id.exposure	id.outcome	outcome	exposure	method	nsnp	b	se	pval	lo_ci	up_ci	or	or_lci95	or_uci95	Exposure_ID
viyN9N	dMRh3L	outcome	exposure	Simple mode	12	-0.00286	0.009306	0.764676	-0.0211	0.015384	0.997148	0.979126	1.015503	GCST90257073
viyN9N	dMRh3L	outcome	exposure	Weighted mode	12	-0.00657	0.00807	0.432931	-0.02238	0.009248	0.993453	0.977864	1.009291	GCST90257073
smud3L	raYOWr	outcome	exposure	MR Egger	17	-0.02412	0.012387	0.070499	-0.0484	0.00016	0.97617	0.952755	1.00016	GCST90257074
smud3L	raYOWr	outcome	exposure	Weighted median	17	-0.00829	0.004482	0.064405	-0.01707	0.000496	0.991745	0.983071	1.000496	GCST90257074
smud3L	raYOWr	outcome	exposure	Inverse variance weighted	17	-0.00589	0.003781	0.119333	-0.0133	0.001522	0.994128	0.986788	1.001523	GCST90257074
smud3L	raYOWr	outcome	exposure	Simple mode	17	-0.0064	0.007339	0.396081	-0.02079	0.007985	0.99362	0.979429	1.008017	GCST90257074
smud3L	raYOWr	outcome	exposure	Weighted mode	17	-0.00718	0.005987	0.247848	-0.01892	0.004554	0.992845	0.981262	1.004565	GCST90257074
hXKneM	j7IwwS	outcome	exposure	MR Egger	5	0.071649	0.03108	0.104479	0.010732	0.132565	1.074278	1.01079	1.141753	GCST90257075
hXKneM	j7IwwS	outcome	exposure	Weighted median	5	0.031057	0.01836	0.09073	-0.00493	0.067043	1.031545	0.995084	1.069342	GCST90257075
hXKneM	j7IwwS	outcome	exposure	Inverse variance weighted	5	0.025909	0.017849	0.146613	-0.00907	0.060893	1.026248	0.990967	1.062785	GCST90257075
hXKneM	j7IwwS	outcome	exposure	Simple mode	5	-0.02413	0.047468	0.637929	-0.11717	0.068905	0.976156	0.889433	1.071335	GCST90257075
hXKneM	j7IwwS	outcome	exposure	Weighted mode	5	0.047289	0.019838	0.075683	0.008406	0.086173	1.048425	1.008441	1.089995	GCST90257075
V51Nk5	iaDgF2	outcome	exposure	MR Egger	9	0.197432	0.119034	0.141155	-0.03587	0.430739	1.218271	0.964762	1.538393	GCST90257076
V51Nk5	iaDgF2	outcome	exposure	Weighted median	9	0.013543	0.028128	0.630181	-0.04159	0.068674	1.013635	0.959265	1.071087	GCST90257076
V51Nk5	iaDgF2	outcome	exposure	Inverse variance weighted	9	-0.00446	0.029468	0.879577	-0.06222	0.053293	0.995545	0.939674	1.054739	GCST90257076
V51Nk5	iaDgF2	outco	exposu	Simple mode	9	0.03399	0.0567	0.5659	-	0.1452	1.0345	0.9256	1.1563	GCST90257

id.exposure	id.outcome	outcome	exposure	method	nsnp	b	se	pval	lo_ci	up_ci	or	or_lci95	or_uci95	Exposure_ID
		me	re			4	78	23	0.07729	79	78	21	62	076
V51Nk5	iaDgF2	outcome	exposure	Weighted mode	9	0.054913	0.05376	0.336936	-0.05046	0.160283	1.056449	0.950795	1.173843	GCST90257076
Mq82WT	B1eFz0	outcome	exposure	MR Egger	9	-0.04637	0.041285	0.298367	-0.12729	0.034545	0.954684	0.880475	1.035149	GCST90257077
Mq82WT	B1eFz0	outcome	exposure	Weighted median	9	-0.04591	0.017051	0.007096	-0.07933	-0.01249	0.955132	0.923739	0.987591	GCST90257077
Mq82WT	B1eFz0	outcome	exposure	Inverse variance weighted	9	-0.03479	0.020812	0.094612	-0.07558	0.006003	0.96581	0.927205	1.006021	GCST90257077
Mq82WT	B1eFz0	outcome	exposure	Simple mode	9	-0.01981	0.031667	0.549113	-0.08187	0.042262	0.98039	0.92139	1.043167	GCST90257077
Mq82WT	B1eFz0	outcome	exposure	Weighted mode	9	-0.04398	0.018208	0.042151	-0.07967	-0.00829	0.956973	0.923422	0.991742	GCST90257077
v206p9	2y1Wyl	outcome	exposure	MR Egger	6	0.042573	0.030238	0.23191	-0.01669	0.10184	1.043493	0.983446	1.107206	GCST90257078
v206p9	2y1Wyl	outcome	exposure	Weighted median	6	0.0423	0.016031	0.008322	0.01088	0.07372	1.043207	1.010939	1.076506	GCST90257078
v206p9	2y1Wyl	outcome	exposure	Inverse variance weighted	6	0.045581	0.016087	0.004607	0.01405	0.077112	1.046636	1.014149	1.080163	GCST90257078
v206p9	2y1Wyl	outcome	exposure	Simple mode	6	0.089994	0.036456	0.056629	0.018541	0.161448	1.094168	1.018714	1.175211	GCST90257078
v206p9	2y1Wyl	outcome	exposure	Weighted mode	6	0.043952	0.016775	0.047091	0.011073	0.07683	1.044932	1.011135	1.079859	GCST90257078
jRfHWb	tNo9iX	outcome	exposure	MR Egger	5	-0.05519	0.083097	0.55404	-0.21806	0.107675	0.946301	0.804073	1.113686	GCST90257079
jRfHWb	tNo9iX	outcome	exposure	Weighted median	5	-0.04721	0.019193	0.013899	-0.08483	-0.00959	0.953885	0.918667	0.990452	GCST90257079
jRfHWb	tNo9iX	outco	exposu	Inverse variance	5	-	0.0364	0.5114	-0.0953	0.0474	0.9763	0.9091	1.0486	GCST90257

id.exposure	id.outcome	outcome	exposure	method	nsnp	b	se	pval	lo_ci	up_ci	or	or_lci95	or_uci95	Exposure_ID
		me	re	weighted		0.02391	21	35		7	7	02	15	079
jRfHWb	tNo9iX	outcome	exposure	Simple mode	5	-0.05862	0.044739	0.260276	-0.14631	0.029068	0.943065	0.863891	1.029494	GCST90257079
jRfHWb	tNo9iX	outcome	exposure	Weighted mode	5	-0.04993	0.018933	0.057751	-0.08704	-0.01282	0.951297	0.916644	0.987261	GCST90257079
abTM4M	Mcf244	outcome	exposure	MR Egger	7	0.061621	0.031918	0.111402	-0.00094	0.124179	1.063559	0.999063	1.132219	GCST90257080
abTM4M	Mcf244	outcome	exposure	Weighted median	7	0.036956	0.016157	0.022172	0.00529	0.068623	1.037648	1.005304	1.071033	GCST90257080
abTM4M	Mcf244	outcome	exposure	Inverse variance weighted	7	0.025799	0.018318	0.159021	-0.0101	0.061703	1.026135	0.989946	1.063647	GCST90257080
abTM4M	Mcf244	outcome	exposure	Simple mode	7	0.040416	0.037707	0.324993	-0.03349	0.114321	1.041244	0.967066	1.121112	GCST90257080
abTM4M	Mcf244	outcome	exposure	Weighted mode	7	0.041273	0.015641	0.038602	0.010617	0.071928	1.042136	1.010673	1.074578	GCST90257080
XkVP9n	bQl3sD	outcome	exposure	MR Egger	11	-0.00248	0.026204	0.92667	-0.05384	0.04888	0.997523	0.947584	1.050094	GCST90257081
XkVP9n	bQl3sD	outcome	exposure	Weighted median	11	-0.0089	0.009897	0.368631	-0.0283	0.010501	0.991141	0.9721	1.010556	GCST90257081
XkVP9n	bQl3sD	outcome	exposure	Inverse variance weighted	11	-0.01317	0.008002	0.099851	-0.02885	0.002516	0.986919	0.971561	1.002519	GCST90257081
XkVP9n	bQl3sD	outcome	exposure	Simple mode	11	-0.00632	0.016308	0.706477	-0.03828	0.025644	0.9937	0.96244	1.025975	GCST90257081
XkVP9n	bQl3sD	outcome	exposure	Weighted mode	11	-0.00469	0.018115	0.801133	-0.04019	0.03082	0.995325	0.960605	1.0313	GCST90257081
u5YgNu	oD59RB	outcome	exposure	MR Egger	5	-0.10793	0.088437	0.309488	-0.28127	0.065407	0.897691	0.754828	1.067594	GCST90257082
u5YgNu	oD59RB	outcome	exposure	Weighted median	5	-0.02782	0.028411	0.327534	-0.0835	0.027869	0.972566	0.919887	1.028261	GCST90257082
u5YgNu	oD59RB	outco	exposu	Inverse variance	5	-	0.0227	0.3633	-	0.0238	0.9795	0.9368	1.0241	GCST90257

id.exposure	id.outcome	outcome	exposure	method	nsnp	b	se	pval	lo_ci	up_ci	or	or_lci95	or_uci95	Exposure_ID
		me	re	weighted		0.02066	36	97	0.06523	97	47	55	85	082
u5YgNu	oD59RB	outcome	exposure	Simple mode	5	-0.03464	0.041504	0.450885	-0.11599	0.046709	0.965953	0.890485	1.047817	GCST90257082
u5YgNu	oD59RB	outcome	exposure	Weighted mode	5	-0.0415	0.039123	0.348635	-0.11818	0.035184	0.959352	0.888538	1.035811	GCST90257082
5DK5d7	dUKfpd	outcome	exposure	MR Egger	10	-0.0196	0.04676	0.68609	-0.11125	0.072047	0.980588	0.894713	1.074706	GCST90257083
5DK5d7	dUKfpd	outcome	exposure	Weighted median	10	0.001646	0.02013	0.934842	-0.03781	0.041101	1.001647	0.962896	1.041957	GCST90257083
5DK5d7	dUKfpd	outcome	exposure	Inverse variance weighted	10	-0.00162	0.014674	0.912199	-0.03038	0.027144	0.998383	0.970077	1.027516	GCST90257083
5DK5d7	dUKfpd	outcome	exposure	Simple mode	10	0.002704	0.034484	0.939213	-0.06488	0.070293	1.002708	0.937176	1.072822	GCST90257083
5DK5d7	dUKfpd	outcome	exposure	Weighted mode	10	0.004372	0.02749	0.877154	-0.04951	0.058253	1.004381	0.951696	1.059983	GCST90257083
9AZgcI	tqDBvS	outcome	exposure	MR Egger	11	-0.0125	0.069047	0.860395	-0.14783	0.122836	0.987582	0.86258	1.130699	GCST90257084
9AZgcI	tqDBvS	outcome	exposure	Weighted median	11	0.002138	0.021597	0.921142	-0.04019	0.044468	1.00214	0.960605	1.045471	GCST90257084
9AZgcI	tqDBvS	outcome	exposure	Inverse variance weighted	11	0.001379	0.019832	0.944569	-0.03749	0.04025	1.00138	0.963202	1.041071	GCST90257084
9AZgcI	tqDBvS	outcome	exposure	Simple mode	11	0.012704	0.034387	0.719508	-0.0547	0.080103	1.012785	0.946774	1.083399	GCST90257084
9AZgcI	tqDBvS	outcome	exposure	Weighted mode	11	0.008566	0.027497	0.761804	-0.04533	0.06246	1.008603	0.955684	1.064451	GCST90257084
vxSEHh	9vaHpU	outcome	exposure	MR Egger	8	0.06312	0.069946	0.401611	-0.07397	0.200215	1.065155	0.928696	1.221665	GCST90257085
vxSEHh	9vaHpU	outcome	exposure	Weighted median	8	0.029128	0.023737	0.219773	-0.0174	0.075653	1.029557	0.982754	1.078588	GCST90257085
vxSEHh	9vaHpU	outcome	exposure	Inverse variance weighted	8	0.021913	0.017794	0.218154	-0.01296	0.056789	1.022154	0.98712	1.058432	GCST90257085

id.exposure	id.outcome	outcome	exposure	method	nsnp	b	se	pval	lo_ci	up_ci	or	or_lci95	or_uci95	Exposure_ID
vxSEHh	9vaHpU	outcome	exposure	Simple mode	8	0.055413	0.036656	0.174366	-0.01643	0.127258	1.056977	0.983702	1.13571	GCST90257085
vxSEHh	9vaHpU	outcome	exposure	Weighted mode	8	0.047527	0.029974	0.156855	-0.01122	0.106277	1.048674	0.98884	1.112129	GCST90257085
lG2OyH	sBLDLM	outcome	exposure	MR Egger	8	0.04183	0.095742	0.677469	-0.14582	0.229484	1.042717	0.864309	1.257951	GCST90257086
lG2OyH	sBLDLM	outcome	exposure	Weighted median	8	0.00405	0.025128	0.871942	-0.0452	0.053301	1.004059	0.955806	1.054748	GCST90257086
lG2OyH	sBLDLM	outcome	exposure	Inverse variance weighted	8	0.01798	0.020203	0.373473	-0.02162	0.057578	1.018143	0.978615	1.059267	GCST90257086
lG2OyH	sBLDLM	outcome	exposure	Simple mode	8	-0.00418	0.033787	0.905125	-0.0704	0.062047	0.995833	0.932024	1.064012	GCST90257086
lG2OyH	sBLDLM	outcome	exposure	Weighted mode	8	-0.00062	0.034482	0.986209	-0.0682	0.066968	0.999383	0.934071	1.069261	GCST90257086
8zKBTh	UTmTWQ	outcome	exposure	MR Egger	12	-0.00222	0.021466	0.919623	-0.0443	0.039853	0.997781	0.956671	1.040657	GCST90257087
8zKBTh	UTmTWQ	outcome	exposure	Weighted median	12	0.009027	0.007529	0.230543	-0.00573	0.023783	1.009068	0.994287	1.024068	GCST90257087
8zKBTh	UTmTWQ	outcome	exposure	Inverse variance weighted	12	0.014705	0.005443	0.0069	0.004037	0.025374	1.014814	1.004045	1.025698	GCST90257087
8zKBTh	UTmTWQ	outcome	exposure	Simple mode	12	0.00225	0.013994	0.875169	-0.02518	0.029679	1.002253	0.975136	1.030124	GCST90257087
8zKBTh	UTmTWQ	outcome	exposure	Weighted mode	12	0.000699	0.012419	0.956134	-0.02364	0.02504	1.000699	0.976635	1.025356	GCST90257087
gr7Gzn	KCmTE4	outcome	exposure	MR Egger	8	-0.0141	0.020688	0.520833	-0.05465	0.026444	0.985995	0.946815	1.026797	GCST90257088
gr7Gzn	KCmTE4	outcome	exposure	Weighted median	8	-0.00119	0.005055	0.814612	-0.01109	0.008723	0.998815	0.988968	1.008761	GCST90257088
gr7Gzn	KCmTE4	outcome	exposure	Inverse variance weighted	8	0.002115	0.005454	0.698137	-0.00857	0.012805	1.002118	0.991462	1.012888	GCST90257088
gr7Gzn	KCmTE	outco	exposu	Simple mode	8	-	0.0064	0.4932	-	0.0079	0.9953	0.9829	1.0079	GCST90257

id.exposure	id.outcome	outcome	exposure	method	nsnp	b	se	pval	lo_ci	up_ci	or	or_lci95	or_uci95	Exposure_ID
	4	me	re			0.00463	05	63	0.01718	25	81	64	56	088
gr7Gzn	KCmTE4	outcome	exposure	Weighted mode	8	-0.00378	0.005575	0.519633	-0.01471	0.007148	0.996228	0.985402	1.007173	GCST90257088
pCWKsT	1Z7RJe	outcome	exposure	MR Egger	9	0.006807	0.013858	0.638321	-0.02035	0.033967	1.00683	0.979851	1.034551	GCST90257089
pCWKsT	1Z7RJe	outcome	exposure	Weighted median	9	-0.00268	0.004376	0.540472	-0.01126	0.005899	0.997325	0.988806	1.005916	GCST90257089
pCWKsT	1Z7RJe	outcome	exposure	Inverse variance weighted	9	-0.00304	0.003347	0.364394	-0.0096	0.003524	0.996969	0.99045	1.00353	GCST90257089
pCWKsT	1Z7RJe	outcome	exposure	Simple mode	9	-0.00368	0.006528	0.588859	-0.01647	0.00912	0.996331	0.983664	1.009162	GCST90257089
pCWKsT	1Z7RJe	outcome	exposure	Weighted mode	9	-0.00188	0.005963	0.761217	-0.01356	0.009812	0.998127	0.986529	1.009861	GCST90257089
V779L1	kfTiVO	outcome	exposure	MR Egger	14	-0.00741	0.020809	0.728111	-0.04819	0.033381	0.992622	0.952951	1.033944	GCST90257090
V779L1	kfTiVO	outcome	exposure	Weighted median	14	0.003179	0.005179	0.53939	-0.00697	0.013331	1.003184	0.993051	1.01342	GCST90257090
V779L1	kfTiVO	outcome	exposure	Inverse variance weighted	14	0.000563	0.004718	0.905065	-0.00869	0.009811	1.000563	0.991352	1.009859	GCST90257090
V779L1	kfTiVO	outcome	exposure	Simple mode	14	0.011272	0.010104	0.284802	-0.00853	0.031076	1.011336	0.991504	1.031564	GCST90257090
V779L1	kfTiVO	outcome	exposure	Weighted mode	14	0.008572	0.009375	0.377158	-0.0098	0.026946	1.008609	0.990246	1.027313	GCST90257090
2nyYtU	kATEKG	outcome	exposure	MR Egger	12	0.014094	0.017651	0.443142	-0.0205	0.048691	1.014194	0.979706	1.049895	GCST90257091
2nyYtU	kATEKG	outcome	exposure	Weighted median	12	-0.00586	0.005842	0.315894	-0.01731	0.005592	0.994158	0.982838	1.005607	GCST90257091
2nyYtU	kATEKG	outcome	exposure	Inverse variance weighted	12	-0.0066	0.005281	0.211052	-0.01696	0.003746	0.993417	0.983187	1.003753	GCST90257091
2nyYtU	kATEKG	outcome	exposure	Simple mode	12	-0.01611	0.010324	0.147024	-0.03634	0.004129	0.984022	0.96431	1.004137	GCST90257091

id.exposure	id.outcome	outcome	exposure	method	nsnp	b	se	pval	lo_ci	up_ci	or	or_lci95	or_uci95	Exposure_ID
2nyYtU	kATEKG	outcome	exposure	Weighted mode	12	0.000962	0.009696	0.922787	-0.01804	0.019965	1.000962	0.98212	1.020166	GCST90257091
120NIP	1ylAXU	outcome	exposure	MR Egger	13	-0.00489	0.014321	0.739011	-0.03296	0.023176	0.995118	0.967575	1.023446	GCST90257092
120NIP	1ylAXU	outcome	exposure	Weighted median	13	0.001388	0.005681	0.80703	-0.00975	0.012523	1.001389	0.9903	1.012602	GCST90257092
120NIP	1ylAXU	outcome	exposure	Inverse variance weighted	13	-0.00449	0.004776	0.346664	-0.01386	0.004866	0.995516	0.98624	1.004878	GCST90257092
120NIP	1ylAXU	outcome	exposure	Simple mode	13	0.004205	0.009594	0.668936	-0.0146	0.023009	1.004214	0.985507	1.023276	GCST90257092
120NIP	1ylAXU	outcome	exposure	Weighted mode	13	0.003719	0.008014	0.650916	-0.01199	0.019426	1.003726	0.988083	1.019616	GCST90257092
pMjku0	LZETtZ	outcome	exposure	MR Egger	7	-0.12947	0.106517	0.278428	-0.33825	0.079301	0.878559	0.71302	1.08253	GCST90257093
pMjku0	LZETtZ	outcome	exposure	Weighted median	7	-0.01464	0.02493	0.556979	-0.06351	0.034221	0.985464	0.938469	1.034813	GCST90257093
pMjku0	LZETtZ	outcome	exposure	Inverse variance weighted	7	-0.01146	0.028264	0.685224	-0.06685	0.043941	0.988609	0.935331	1.044921	GCST90257093
pMjku0	LZETtZ	outcome	exposure	Simple mode	7	-0.04951	0.040453	0.266858	-0.1288	0.029777	0.951694	0.87915	1.030224	GCST90257093
pMjku0	LZETtZ	outcome	exposure	Weighted mode	7	-0.01636	0.029001	0.593037	-0.07321	0.040479	0.983769	0.929409	1.041309	GCST90257093
tL6upM	iqAlxR	outcome	exposure	MR Egger	6	-0.16173	0.141489	0.316773	-0.43905	0.115586	0.850668	0.644647	1.122531	GCST90257094
tL6upM	iqAlxR	outcome	exposure	Weighted median	6	0.012204	0.028622	0.669814	-0.04389	0.068303	1.012279	0.957055	1.07069	GCST90257094
tL6upM	iqAlxR	outcome	exposure	Inverse variance weighted	6	0.011004	0.022812	0.629536	-0.03371	0.055716	1.011065	0.966854	1.057298	GCST90257094
tL6upM	iqAlxR	outcome	exposure	Simple mode	6	-0.00511	0.042979	0.909954	-0.08935	0.079126	0.994901	0.914525	1.082341	GCST90257094
tL6upM	iqAlxR	outco	exposu	Weighted mode	6	0.00457	0.0388	0.9109	-	0.0807	1.0045	0.9309	1.0840	GCST90257

id.exposure	id.outcome	outcome	exposure	method	nsnp	b	se	pval	lo_ci	up_ci	or	or_lci95	or_uci95	Exposure_ID
		me	re				46	26	0.07157	08	81	33	54	094
WpSDil	Ww6R2d	outcome	exposure	MR Egger	5	0.004856	0.031727	0.888067	-0.05733	0.06704	1.004868	0.944284	1.069339	GCST90257095
WpSDil	Ww6R2d	outcome	exposure	Weighted median	5	0.006889	0.012369	0.577569	-0.01735	0.031131	1.006912	0.982796	1.031621	GCST90257095
WpSDil	Ww6R2d	outcome	exposure	Inverse variance weighted	5	0.002444	0.009974	0.806453	-0.0171	0.021992	1.002447	0.983041	1.022235	GCST90257095
WpSDil	Ww6R2d	outcome	exposure	Simple mode	5	0.009014	0.01681	0.620233	-0.02393	0.041961	1.009055	0.976351	1.042854	GCST90257095
WpSDil	Ww6R2d	outcome	exposure	Weighted mode	5	0.010097	0.015854	0.558855	-0.02098	0.041171	1.010148	0.979241	1.04203	GCST90257095
52s5Jb	LVSjTk	outcome	exposure	MR Egger	8	0.05201	0.088556	0.57841	-0.12156	0.22558	1.053386	0.885538	1.253049	GCST90257096
52s5Jb	LVSjTk	outcome	exposure	Weighted median	8	0.002771	0.020312	0.891489	-0.03704	0.042582	1.002775	0.963638	1.043501	GCST90257096
52s5Jb	LVSjTk	outcome	exposure	Inverse variance weighted	8	0.00088	0.017575	0.960085	-0.03357	0.035327	1.00088	0.966989	1.035958	GCST90257096
52s5Jb	LVSjTk	outcome	exposure	Simple mode	8	0.003157	0.032688	0.925759	-0.06091	0.067226	1.003162	0.940907	1.069537	GCST90257096
52s5Jb	LVSjTk	outcome	exposure	Weighted mode	8	-0.00294	0.024604	0.908262	-0.05116	0.045284	0.997065	0.950124	1.046325	GCST90257096
0klSPj	ckh7jD	outcome	exposure	MR Egger	6	0.023089	0.02095	0.332266	-0.01797	0.06415	1.023358	0.982188	1.066253	GCST90257097
0klSPj	ckh7jD	outcome	exposure	Weighted median	6	0.00028	0.006714	0.966725	-0.01288	0.013439	1.00028	0.987203	1.01353	GCST90257097
0klSPj	ckh7jD	outcome	exposure	Inverse variance weighted	6	-0.00569	0.005015	0.256168	-0.01552	0.004135	0.994322	0.984597	1.004143	GCST90257097
0klSPj	ckh7jD	outcome	exposure	Simple mode	6	0.001429	0.010301	0.895103	-0.01876	0.021618	1.00143	0.981414	1.021853	GCST90257097
0klSPj	ckh7jD	outcome	exposure	Weighted mode	6	0.001331	0.008621	0.88332	-0.01557	0.018228	1.001332	0.984555	1.018396	GCST90257097

id.exposure	id.outcome	outcome	exposure	method	nsnp	b	se	pval	lo_ci	up_ci	or	or_lci95	or_uci95	Exposure_ID
tLTdFm	LD2gv3	outcome	exposure	MR Egger	7	0.005651	0.011052	0.630941	-0.01601	0.027313	1.005667	0.984116	1.027689	GCST90257098
tLTdFm	LD2gv3	outcome	exposure	Weighted median	7	-0.00137	0.004394	0.754442	-0.00999	0.007237	0.998627	0.990064	1.007263	GCST90257098
tLTdFm	LD2gv3	outcome	exposure	Inverse variance weighted	7	0.001229	0.003326	0.711841	-0.00529	0.007748	1.001229	0.994723	1.007778	GCST90257098
tLTdFm	LD2gv3	outcome	exposure	Simple mode	7	-0.00382	0.006503	0.577934	-0.01657	0.008922	0.996183	0.983566	1.008962	GCST90257098
tLTdFm	LD2gv3	outcome	exposure	Weighted mode	7	-0.00393	0.006035	0.53912	-0.01576	0.0079	0.996078	0.984365	1.007931	GCST90257098
9p5FnV	3mXriG	outcome	exposure	MR Egger	9	0.127024	0.079817	0.155535	-0.02942	0.283465	1.135445	0.971012	1.327723	GCST90257099
9p5FnV	3mXriG	outcome	exposure	Weighted median	9	-0.00609	0.024099	0.800538	-0.05332	0.041145	0.99393	0.948075	1.042003	GCST90257099
9p5FnV	3mXriG	outcome	exposure	Inverse variance weighted	9	-0.0093	0.026762	0.72823	-0.06175	0.043155	0.990744	0.940114	1.0441	GCST90257099
9p5FnV	3mXriG	outcome	exposure	Simple mode	9	-0.0162	0.038594	0.685637	-0.09185	0.05944	0.983927	0.912244	1.061242	GCST90257099
9p5FnV	3mXriG	outcome	exposure	Weighted mode	9	-0.00202	0.034782	0.955106	-0.07019	0.066152	0.997982	0.932214	1.068389	GCST90257099
ghAM97	pAqq1J	outcome	exposure	MR Egger	3	-0.01238	0.019782	0.644105	-0.05115	0.026397	0.9877	0.950137	1.026748	GCST90257100
ghAM97	pAqq1J	outcome	exposure	Weighted median	3	-0.00645	0.005454	0.236734	-0.01714	0.004237	0.993568	0.983004	1.004246	GCST90257100
ghAM97	pAqq1J	outcome	exposure	Inverse variance weighted	3	-0.00184	0.006804	0.787049	-0.01517	0.011498	0.998164	0.98494	1.011564	GCST90257100
ghAM97	pAqq1J	outcome	exposure	Simple mode	3	-0.00753	0.007397	0.416033	-0.02202	0.006973	0.992503	0.978216	1.006998	GCST90257100
ghAM97	pAqq1J	outcome	exposure	Weighted mode	3	-0.00699	0.005693	0.344175	-0.01815	0.004164	0.99303	0.982012	1.004172	GCST90257100
g2LqpK	XAjQY	outco	exposu	MR Egger	6	0.06135	0.0266	0.0823	0.00921	0.1134	1.0632	1.0092	1.1201	GCST90257

id.exposure	id.outcome	outcome	exposure	method	nsnp	b	se	pval	lo_ci	up_ci	or	or_lci95	or_uci95	Exposure_ID
	A	me	re			6	02	53	6	96	78	59	88	101
g2LqpK	XAjQYA	outcome	exposure	Weighted median	6	0.04135	0.017402	0.017495	0.007242	0.075458	1.042217	1.007268	1.078378	GCST90257101
g2LqpK	XAjQYA	outcome	exposure	Inverse variance weighted	6	0.030089	0.014399	0.036647	0.001867	0.058311	1.030546	1.001869	1.060045	GCST90257101
g2LqpK	XAjQYA	outcome	exposure	Simple mode	6	0.023292	0.032682	0.507909	-0.04077	0.08735	1.023566	0.960054	1.091279	GCST90257101
g2LqpK	XAjQYA	outcome	exposure	Weighted mode	6	0.042388	0.017748	0.062523	0.007601	0.077174	1.043299	1.00763	1.08023	GCST90257101
KxDPuL	nn5T1r	outcome	exposure	MR Egger	10	-0.00181	0.010017	0.861006	-0.02144	0.017821	0.99819	0.978785	1.017981	GCST90257102
KxDPuL	nn5T1r	outcome	exposure	Weighted median	10	-0.00077	0.004262	0.85753	-0.00912	0.007588	0.999235	0.990924	1.007617	GCST90257102
KxDPuL	nn5T1r	outcome	exposure	Inverse variance weighted	10	0.001561	0.003125	0.617431	-0.00456	0.007687	1.001562	0.995446	1.007716	GCST90257102
KxDPuL	nn5T1r	outcome	exposure	Simple mode	10	-0.00217	0.007067	0.765738	-0.01602	0.01168	0.997832	0.984107	1.011749	GCST90257102
KxDPuL	nn5T1r	outcome	exposure	Weighted mode	10	-0.00242	0.006457	0.716295	-0.01508	0.010234	0.997581	0.985036	1.010286	GCST90257102
ggnnpt	8gXhkg	outcome	exposure	MR Egger	10	-0.00025	0.011222	0.982468	-0.02225	0.021741	0.999746	0.977996	1.021979	GCST90257103
ggnnpt	8gXhkg	outcome	exposure	Weighted median	10	0.006009	0.003345	0.072427	-0.00055	0.012565	1.006027	0.999453	1.012644	GCST90257103
ggnnpt	8gXhkg	outcome	exposure	Inverse variance weighted	10	0.00495	0.002611	0.057983	-0.00017	0.010068	1.004963	0.999832	1.010119	GCST90257103
ggnnpt	8gXhkg	outcome	exposure	Simple mode	10	0.002433	0.005264	0.654963	-0.00789	0.01275	1.002436	0.992146	1.012832	GCST90257103
ggnnpt	8gXhkg	outcome	exposure	Weighted mode	10	0.005178	0.00488	0.316284	-0.00439	0.014742	1.005191	0.995623	1.014851	GCST90257103
6SQp1g	7TcqHQ	outcome	exposure	MR Egger	8	-0.00114	0.024846	0.964832	-0.04984	0.047557	0.998859	0.951381	1.048706	GCST90257104

id.exposure	id.outcome	outcome	exposure	method	nsnp	b	se	pval	lo_ci	up_ci	or	or_lci95	or_uci95	Exposure_ID
6SQp1g	7TcqHQ	outcome	exposure	Weighted median	8	-0.00269	0.010328	0.79454	-0.02293	0.017554	0.997314	0.977328	1.017708	GCST90257104
6SQp1g	7TcqHQ	outcome	exposure	Inverse variance weighted	8	-0.00531	0.007878	0.500299	-0.02075	0.010131	0.994704	0.979462	1.010183	GCST90257104
6SQp1g	7TcqHQ	outcome	exposure	Simple mode	8	-0.00159	0.015806	0.922712	-0.03257	0.02939	0.998412	0.967956	1.029826	GCST90257104
6SQp1g	7TcqHQ	outcome	exposure	Weighted mode	8	-0.00159	0.015795	0.922657	-0.03255	0.029368	0.998412	0.967977	1.029803	GCST90257104
P0IfT6	7Ej6UL	outcome	exposure	MR Egger	8	0.074017	0.108733	0.521434	-0.1391	0.287133	1.076825	0.870142	1.332602	GCST90257105
P0IfT6	7Ej6UL	outcome	exposure	Weighted median	8	0.017754	0.027804	0.523116	-0.03674	0.07225	1.017913	0.963925	1.074924	GCST90257105
P0IfT6	7Ej6UL	outcome	exposure	Inverse variance weighted	8	-0.00233	0.021738	0.914722	-0.04493	0.040278	0.997675	0.956061	1.0411	GCST90257105
P0IfT6	7Ej6UL	outcome	exposure	Simple mode	8	0.027288	0.045395	0.566707	-0.06169	0.116263	1.027664	0.940178	1.123291	GCST90257105
P0IfT6	7Ej6UL	outcome	exposure	Weighted mode	8	0.027828	0.045155	0.557212	-0.06068	0.116332	1.028219	0.941128	1.123369	GCST90257105
